# Metagenomics-resolved genomics provides novel insights into chitin turnover, metabolic specialization, and niche partitioning in the octocoral microbiome

**DOI:** 10.1186/s40168-022-01343-7

**Published:** 2022-09-22

**Authors:** Tina Keller-Costa, Lydia Kozma, Sandra G. Silva, Rodolfo Toscan, Jorge Gonçalves, Asunción Lago-Lestón, Nikos C. Kyrpides, Ulisses Nunes da Rocha, Rodrigo Costa

**Affiliations:** 1 Institute for Bioengineering and Biosciences, Instituto Superior Técnico, University of Lisbon, Lisbon, Portugal; 2 Associate Laboratory i4HB - Institute for Health and Bioeconomy, Instituto Superior Técnico, University of Lisbon, Lisbon, Portugal; 3 École Polytechnique Fédérale de Lausanne, Écublens, Switzerland; 4 Helmholtz Centre for Environmental Research, Leipzig, Germany; 5 Centro de Ciências Do Mar, Universidade Do Algarve, Faro, Portugal; 6 Centro de Investigación Científica Y de Educación Superior de Ensenada, Ensenada, Mexico; 7 Department of Energy, Joint Genome Institute, Lawrence Berkeley National Laboratory, Berkeley, CA USA

**Keywords:** Symbiosis, MAGs, Gorgonians, Facultative anaerobes, Chitinases, Secondary metabolism, *Endozoicomonadaceae*, *Thioglobaceae*, *Mollicutes*

## Abstract

**Background:**

The role of bacterial symbionts that populate octocorals (Cnidaria, Octocorallia) is still poorly understood. To shed light on their metabolic capacities, we examined 66 high-quality metagenome-assembled genomes (MAGs) spanning 30 prokaryotic species, retrieved from microbial metagenomes of three octocoral species and seawater.

**Results:**

Symbionts of healthy octocorals were affiliated with the taxa *Endozoicomonadaceae*, Candidatus *Thioglobaceae*, *Metamycoplasmataceae*, unclassified *Pseudomonadales*, *Rhodobacteraceae*, unclassified *Alphaproteobacteria* and Ca. *Rhabdochlamydiaceae*. Phylogenomics inference revealed that the *Endozoicomonadaceae* symbionts uncovered here represent two species of a novel genus unique to temperate octocorals, here denoted Ca. *Gorgonimonas* *eunicellae* and Ca. *Gorgonimonas* *leptogorgiae*. Their genomes revealed metabolic capacities to thrive under suboxic conditions and high gene copy numbers of serine-threonine protein kinases, type 3-secretion system, type-4 pili, and ankyrin-repeat proteins, suggesting excellent capabilities to colonize, aggregate, and persist inside their host. Contrarily, MAGs obtained from seawater frequently lacked symbiosis-related genes. All *Endozoicomonadaceae* symbionts harbored endo-chitinase and chitin-binging protein-encoding genes, indicating that they can hydrolyze the most abundant polysaccharide in the oceans. Other symbionts, including *Metamycoplasmataceae* and Ca. *Thioglobaceae*, may assimilate the smaller chitin oligosaccharides resulting from chitin breakdown and engage in chitin deacetylation, respectively, suggesting possibilities for substrate cross-feeding and a role for the coral microbiome in overall chitin turnover. We also observed sharp differences in secondary metabolite production potential between symbiotic lineages. Specific *Proteobacteria *taxa may specialize in chemical defense and guard other symbionts, including *Endozoicomonadaceae*, which lack such capacity.

**Conclusion:**

This is the first study to recover MAGs from dominant symbionts of octocorals, including those of so-far unculturable *Endozoicomonadaceae*, Ca. *Thioglobaceae* and *Metamycoplasmataceae* symbionts. We identify a thus-far unanticipated, global role for *Endozoicomonadaceae* symbionts of corals in the processing of chitin, the most abundant natural polysaccharide in the oceans and major component of the natural zoo- and phytoplankton feed of octocorals. We conclude that niche partitioning, metabolic specialization, and adaptation to low oxygen conditions among prokaryotic symbionts likely contribute to the plasticity and adaptability of the octocoral holobiont in changing marine environments. These findings bear implications not only for our understanding of symbiotic relationships in the marine realm but also for the functioning of benthic ecosystems at large.

Video Abstract

**Supplementary Information:**

The online version contains supplementary material available at 10.1186/s40168-022-01343-7.

## Background

Octocorals (Octocorallia, Anthozoa, Cnidaria) are an integral part of benthic marine ecosystems, increasing habitat complexity and biodiversity where they abound [1, 2]. Octocorals differ from scleractinian corals (Hexacorallia) by the eightfold symmetry of their polyps and usually lack a stony skeleton, though their tissue is supported by small, internal skeletal elements (calcite sclerites) [3]. They amount to over 3500 species [4], possessing worldwide distribution from polar over temperate to tropical regions and from shallow waters to the deep sea [2, 5]. Octocorals suspension feed on large quantities of debris and phyto- and zooplankton [6]. They thus play paramount roles in coastal food chains, helping to regulate primary and secondary production [6, 7]. They also form associations with various microorganisms, including micro-eukaryotes, bacteria, archaea, and viruses [reviewed in 7].

Over the past two decades, heat waves and infectious diseases have led to significant mortalities in Mediterranean and northeast Atlantic octocoral populations [8–12]. These mortality events can alter critical ecosystem processes and result in biodiversity loss in the benthos of temperate zones [9]. In other parts of the world, however, octocorals continued to thrive, while scleractinian corals faced a rapid decline from climate change. Studies in the Red Sea, the Caribbean, and the Pacific and Indian Oceans showed reef community shifts from scleractinian corals towards octocorals, increasing octocoral cover from less than 10% to nearly 50% in some regions [reviewed in 7]. It is yet to be determined why some octocorals resist while others are affected by climate change scenarios — but their associated microbiomes likely play a fundamental role in this response.

Owing to advances in high-throughput sequencing and analysis of phylogenetic marker genes (e.g., the 16S rRNA gene), our understanding of the taxonomic composition, diversity, host specificity, geographic variability, and seasonal stability of the octocoral microbiome has increased considerably in the past few years [7, 13–17]. The prokaryotic assemblages of corals, and octocorals, are frequently dominated by *Endozoicomonadaceae *and other *Gammaproteobacteria* phylotypes, in addition to members of *Alphaproteobacteria*, *Mollicutes*, *Flavobacteriia*, *Actinobacteria* and *Spirochaetes* [7, 15, 18, 19]. *Endozoicomonadaceae* symbionts can make up to 96% of a coral prokaryotic community and are generally considered indicators of coral health [7, 15, 20]. Despite their ubiquity and abundance in corals worldwide, less than ten coral-associated *Endozoicomonas* strains (comprising five formally described *Endozoicomonas* species) exist in culture or had their genomes sequenced, as shown by a meta-analysis of over 3050 coral bacterial isolates from more than 80 coral species [21]. Among these, only two cultured *Endozoicomonas* species, *E.* *euniceicola* and *E.* *gorgoniicola*, derive from octocorals [22], and no genome sequence of an octocoral-derived *Endozociomonadaceae* symbiont is yet publicly available. Moreover, recent studies have suggested that cultured *Endozoicomonas* isolates are phylogenetically distant from the dominant, so-far uncultured *Endozoicomonadaceae* phylotypes populating temperate octocorals [13, 15, 19]. Therefore, we still know little about the role of this “core” bacterial family in octocorals and in the functioning of benthic ecosystems at large.

Despite our current, improved view of prokaryotic diversity in corals, knowledge of the functional attributes of coral-associated prokaryotes remains limited, especially regarding the symbionts of octocorals. Collectively, recent omics studies indicate the provision of vitamins and amino acids and participation in nutrient cycling and dimethylsulfoniopropionate metabolization as putative roles of bacterial symbionts of scleractinian corals [21, 23–26]. Comparative genomics of *Alphaproteobacteria*, *Vibrio*, and *Aquimarina* isolates from octocorals further revealed the presence of various biosynthetic gene clusters (SM-BGCs) coding for polyketide, terpene, and antimicrobial peptide production, suggesting an involvement of octocoral bacteria in chemical defense [21, 27–29]. Our recent shotgun metagenomics survey showed that high abundances of eukaryotic-like proteins, exo- and endonucleases, phage lysogenization regulators, and micronutrient acquisition-related genes distinguish the prokaryotic communities of healthy from necrotic octocoral tissue, likely contributing to the stability of the symbiotic microbiome [15]. However, the functional contributions and mechanisms of interaction of microbial symbionts within the octocoral holobiont remain largely unknown.

In this study, we examine metagenome-assembled genomes (MAGs) from 17 shotgun-sequenced microbial metagenomes of three octocoral species (healthy and necrotic *Eunicella* *gazella* tissue, healthy *Eunicella* *verrucosa*, and *Leptogorgia* *sarmentosa*) and surrounding seawater to connect microbial taxonomy with function in a cultivation-independent fashion. We shed light on the likely roles and mechanisms of interaction of dominant bacterial taxa in octocorals. This study is the first to retrieve and compare multiple draft genomes from uncultured symbionts of octocorals, addressing the hypothesis of microbial niche partitioning in the octocoral holobiont.

## Methods

### Metagenome samples

The 17 microbial metagenomes used for the binning of MAGs in this study are publicly available (PRJEB13222) and have been described in our earlier study [15]. Briefly, the samples were collected by scuba diving at 17-m depth on June 17, 2014, in the Atlantic Ocean off the coast of Faro, Algarve, and Portugal (“Pedra da Greta”: Latitude 36° 58′ 47.2 N, Longitude 7° 59′ 20.8 W). Branches (10–20 cm each) of 10 colonies from three octocoral (Alcyonacea, Gorgoniidae) species, *Eunicella* *gazella* (*N* = 6: 3 × healthy, 3 × necrotic; EG15H, EG15N; EG16H, EG16N; EG18H, EG18N), *Eunicella* *verrucosa* (*N* = 4; EV01–EV04), and *Leptogorgia* *sarmentosa* (*N* = 3; LS06–LS08), were sampled. From *E.* *gazella*, branches of both healthy (H) and necrotic (N) tissue were sampled from three independent colonies, each of which displaying both conditions to directly compare the octocoral-associated microbial community in healthy versus necrotic states. The necrotic *E.* *gazella* tissue was characterized by a change of tissue color (from white to brown) and integrity which indicates necrosis and ultimately leads to coenenchyme detachment and loss. Octocoral samples were placed, in situ, separately in Ziploc® bags containing natural seawater. In addition, replicate samples of surrounding seawater (*N* = 4; SW01–SW04; ca 2 L each) were collected ca. 1 m above the corals in separate Ziploc® bags. All samples were transported to the laboratory in a cooling box within 1.5 h post sampling and immediately processed upon arrival. Octocoral branches were washed with artificial seawater to remove exogenous microorganisms and aseptically cut into smaller pieces. The soft tissue (coenenchyme and polyps) was then separated from the inner gorgonin skeleton with a scalpel and homogenized in sterile Ca^2+^ and Mg^2+^-free artificial seawater (CMFASW: 27 g L^−1^ NaCl, 1 g L^−1^ NaSO_4_, 0.8 g L^−1^ KCl and 0.18 g L^−1^ NaHCO_3_, 1 g of soft tissue per 9 mL CMFASW w/v) using a sterile mortar and pestle. The obtained cell homogenates were then subjected to differential centrifugation as described in [19] to retrieve microbial cell pellets. Each seawater sample was filtered through a sterile 0.22 μm nitrocellulose membrane filter (Millipore, Billerica, MA, USA; 47 mm) using a vacuum pump. Microbial pellets and seawater filters were stored at − 80 °C until metagenomic DNA extraction with the UltraClean® Soil DNA isolation kit (MO BIO, Carlsbad, CA, USA) according to the manufacturer’s instructions. Equivalent amounts of DNA per sample biotope (i.e., healthy, and necrotic octocoral tissue, seawater) were sent for next-generation shotgun sequencing on an Illumina HiSeq 2500 device at MR DNA (Shallowater, TX, USA). DNA libraries were prepared for sequencing using the Nextera DNA Sample preparation kit (Illumina) after the manufacturer’s instructions and sequenced paired end with sequence depth calibrated at c. 20 million 101 bp reads per sample.

### Binning of metagenome assembled genomes (MAGs)

Metagenome reads obtained for each sample were assembled using the MetaWRAP v1.0.5 pipeline [30] and encompassed reads quality control with the MetaWRAP galore module, followed by assembly with the meta-SPAdes module 3.13.0 [31]. Eukaryotic contigs were then filtered out of the resulting assemblies using EukRep v.0.6.6 [32], generating individual “prokaryotic-enriched” assembly files per sample [15]. Metagenomic binning was performed in this study on the “prokaryotic-enriched” assemblies using MetaBAT 2 [33], Maxbin 2.0 [34],and CONCOCT [35] within the MetaWRAP binning module. Binning_refiner [36] was used to refine bins and produce a superior bin set within MetaWRAP. Genome completeness, contamination, and strain heterogeneity scores were calculated using CheckM v1.0.11 [37] with default parameters. Following the approach of Parks et al. [38], an overall quality score was calculated for each MAG (completeness — 5 × contamination), and MAGs possessing a quality score above 50% were kept for analysis, resulting in a final dataset of 66 MAGs. We then categorized the 66 MAGs into “high-quality MAGs” (when completeness was above 90% and contamination below 5%) and “medium quality MAGs” (completeness above 50% and contamination below 10%), according to MIMAG guidelines [39]. The Microbial Genome Atlas (MiGA) [40] was used to obtain genome metrics such as number of contigs, genome size, GC content, and N50 values. MiGA (accessed on 25th of November 2021) was also used to identify the closest relative (i.e., genome of a type strain) of each MAG, based on whole-genome average amino acid identity values (AAI%).

### Taxonomic assignment and species-level similarity of MAGs

The Genome Taxonomy Database Toolkit (GTDB-Tk) [41] v.1.5.0 (release 06-RS202) was used to perform taxonomy assignment of all MAGs obtained in this study. High taxonomic rank assignments were afterwards manually curated to comply with the list of prokaryotic names with standing in nomenclature (LPSN) [42, 43] as deemed necessary. FastANI [44] was used to compute whole-genome average nucleotide identity (ANI) percent values in a pairwise fashion whenever two or more MAGs shared the same taxonomic assignment. MAGs which shared ≥ 95% ANI were considered to belong to the same species [44–46].

### Phylogenomics of the *Endozoicomonadaceae* and Ca. *Thioglobaceae* families

Owing to their high frequency across healthy octocoral samples and presumed taxonomic distinctiveness and novelty, we thoroughly explored the phylogenomic relatedness of the 11 *Endozoicomonadaceae *and six Ca. *Thioglobaceae* MAGs of this study with their closest relatives. Two phylogenomic trees were created, one for each family. Details on the genomes used for tree construction are provided in Additional file 1. Both trees were constructed with the SpeciesTreeBuilder v.01.0 application of the DOE Systems Biology Knowledgebase (KBase) [47] using the function “Insert Set of Genomes into Species Tree,” after annotating all isolate genomes, MAGs and SAGs with Prokka [48]. SpeciesTreeBuilder uses the FastTree2 algorithm [49] to infer maximum-likelihood (ML) phylogenies for large alignments. Alignments were based on a set of 49 core genes defined by Clusters of Orthologous Groups of proteins (COG) families. Graphical visualization and editing of the trees were made in iTOL v4 (Interactive Tree Of Life) [50].

### Functional annotation of MAGs

Functional annotation of MAGs encompassed the generation of COG profiles, metabolic pathway reconstruction, and genome mining for secondary metabolite biosynthetic gene clusters (SM-BGCs). COG annotation was performed for all MAGs using our in-house, automated genome annotation pipeline MeLanGE as documented on GitHub (https://sandragodinhosilva.github.io/MeLanGE). Briefly, all MAGs (contig fasta files) were first annotated with Prokka v1.14.6 [48] to obtain GenBank (gbk) format and amino acid fasta files. Thereafter, proteins were queried against the COGs database implemented within NCBI’s Conserved Domain Database (CDD) through Reversed Position Specific Blast (RPS-BLAST) from the BLAST + suite (v2.9.0), and the best hit per ORF, above the cutoff of E 1e-5, was selected. MAGs were further annotated with the RAST server version 2.0 [51, 52] using the RASTtk annotation scheme with default settings and the “build metabolic models” setting activated. Using the “KEGG metabolic analysis” tool within RAST, KEGG metabolic maps were constructed for selected metabolic pathways and individual MAGs (not to model metabolic interactions between species). Complete chitinase coding sequences (CDS) present on the 11 *Endozoicomonadaceae* MAGs were retrieved from RAST and characterized as described in Additional file 1. Identification of SM-BGCs across all MAGs was performed using antiSMASH v5.0 [53] with default parameters and extra features “all on”. The degree of novelty of SM-BGCs was assessed through matches with the Minimum Information about a Biosynthetic Gene cluster (MIBiG) database [54, 55].

### Data analyses and statistics

Multivariate analysis of COG-based functional profiles was carried out on Hellinger-transformed data (i.e., square root of the relative abundance of each COG entry on a MAG). Euclidean distances were then calculated from COG abundance distributions across MAGs, and a principal components analysis (PCA) was performed using PAST v3.25 [56]. One-way permutational analysis of variance (PERMANOVA) was used to test for overall differences in functional profiles among MAGs belonging to different taxonomic orders. To determine COG functions that contributed most to the dissimilarity between MAGs at the order level, similarity percentage analysis (SIMPER) was performed in PAST v 3.25 [84]. The top ten most differentiating COG functions were then plotted as vectors on the PCA diagram to explore relationships between gene functions and symbiont taxonomy. Moreover, Welch’s unequal variances *t*-tests (one sided) were conducted within STAMP v2.0.953 [57] to identify COG entries that were significantly enriched on MAGs affiliated with the *Endozoicomonadaceae* (*N* = 11) and Ca. *Thioglobaceae* (*N* = 6) families. Multiple test correction was performed with the Benjamini–Hochberg method to decrease false discovery rates, and COG entries representing fivefold (Ca. *Thioglobaceae* MAGs) and tenfold (*Endozoicomonadaceae* MAGs) enrichments were selected for further analysis.

## Results

### Dataset overview

The final dataset analyzed in this study comprised 66 MAGs, 65 of which derived from *Bacteria* and one from *Archaea* (Table S1, Additional file 2). Twenty-five MAGs were obtained from healthy octocoral samples (all species together), 14 MAGs from necrotic *E.* *gazella* tissue, and 27 MAGs, including the archaeal one, from seawater. Of the 66 MAGs, 30 and 36 were of high and medium quality, respectively. Genome completeness ranged from 55.03 to 99.86% with an average of 83.57% across the 66 MAGs, while contamination ranged from 0 to 4.64% with an average of 1.16% (Table 1; Table S2, Additional file 2). Average genome size and GC content ranged from only 0.63 Mb and 22.3% in *Metamycoplasmataceae* to 4.2 Mb and 59.6% in Ca. *Inquilinaceae* (*Alphaproteobacteria*), both symbionts of healthy octocorals (Table 1, Table S1, Additional file 2). The mean and median genome size of the 66 MAGs was 2.38 Mb and 2.46 Mb, respectively, whereby genome size was more related with MAG taxonomy than with origin (Table 1; Table S2, Additional file 2).

**Table 1:** Genome features of the MAGs from the 30 prokaryotic species analyzed in this study

Class	Species-level taxonomic affiliation of MAGs	Nª MAGs	Genome size Mb	GC content %	N° of coding Sequences	Complete-ness %	Contami-nation %
*Mollicutes*	*Metamycoplasmataceae* (DT-68 sp.)	3	0.63 ± 0.13	22.33 ± 0.72	685 ± 63	84.07 ± 17.26	0.08 ± 0.14
*Chlamydiia*	Ca. *Rhabdochlamydiaceae* (SZUA-160 sp.)	1	0.88	44.38	1015	60.07	0.00
*Gammaproteobacteria*	Ca. *Gorgonimonas eunicellae*	7	2.79 ± 0.13	29.68 ± 0.05	2177 ± 121	73.17 ± 1.38	1.52 ± 0.96
	Ca. *Gorgonimonas* *leptogorgiae*	4	3.15 ± 0.60	29.77 ± 0.29	2603 ± 355	70.20 ± 4.15	1.06 ± 0.40
	Ca. *Thiocorallibacter* *gorgonii*	2	1.41 ± 0.16	29.19 ± 0.17	2612 ± 6	90.95 ± 6.72	1.24 ± 0.35
	Ca. *Microaerophilica* *antagonistica*	4	1.85 ± 0.12	37.47 ± 0.07	1763 ± 101	93.05 ± 2.33	0.82 ± 0.50
	*Pseudomonadales* (DT-91 sp.)	2	3.29 ± 0.52	40.98 ± 0.09	3505 ± 240	77.02 ± 18.70	1.35 ± 1.90
	*Cardiobacteriales* sp.	2	2.50 ± 0.20	42.72 ± 0.05	2560 ± 145	85.7 0 ± 6.34	0.57 ± 0.00
	*Luminiphilus* sp009886815	3	3.19 ± 0.48	52.79 ± 0.19	3307 ± 580	85.67 ± 10.49	3.39 ± 1.10
*Alphaproteobacteria*	*Ruegeria* sp. (EG16H_Bin1)	1	2.49	56.18	3405	55.03	0.38
	*Ruegeria* sp900313035	1	4.16	56.55	4564	87.01	1.38
	*Yoonia* sp. (EG16N_Bin1)	1	3.62	56.98	3750	99.39	0.38
	*Yoonia* sp. (EG16N_Bin5)	1	2.69	53.45	2878	95.52	1.22
	*Yoonia* sp. (EV01H_Bin2)	1	3.05	57.15	3798	83.28	1.47
	*Aliiroseovarius* sp. (EG18N_Bin4)	1	2.06	51.31	2606	70.61	1.13
	*Planktomarina* sp002683685	4	2.28 ± 0.26	54.87 ± 0.07	2392 ± 105	93.47 ± 7.83	0.87 ± 0.70
	*Amylibacter* sp900197625	4	1.70 ± 0.27	37.10 ± 0.70	1855 ± 183	75.16 ± 14.49	1.54 ± 1.37
	*Rhodobacteraceae* (LGRT01 sp001642945)	3	2.91 ± 0.05	51.36 ± 0.05	2903 ± 27	96.36 ± 0.81	0.36 ± 0.19
	Ca. *Parvibaculales* (UBA8337 sp900197605)	2	1.35 ± 0.03	54.77 ± 0.02	1646 ± 37	80.11 ± 3.25	1.74 ± 0.00
	*Lentilitoribacter* sp900537175	1	3.63	44.39	3580	97.27	0.52
	Unclassified *Rhizobiaceae* (EG18N_Bin5)	1	2.79	58.16	2656	98.96	0.08
	Unclassified *Rhizobiaceae* (EG18N_Bin7)	1	2.58	41.22	2613	93.88	0.2
	Ca. *Inquilinaceae* (JAAAOG01 sp.)	1	4.23	59.61	5446	68.01	0.44
	Unclassified *Alphaproteobacteria*	2	1.67 ± 0.20	50.03 ± 0.16	1668 ± 224	89.25 ± 4.56	0.81 ± 0.38
*Flavobacteriia*	*Aquimarina* sp. (EG16N_Bin3)	1	3.83	32.22	3501	93.00	0.67
	*Flavobacteriaceae* MS024-2A sp002167945	1	1.49	37.84	1570	66.19	2.73
	*Flavobacteriaceae* (MS024-2A sp002292265)	1	1.39	41.03	1492	74.42	2.26
	*Schleiferiaceae* (UBA10364 sp002387615)	4	1.83 ± 0.07	44.29 ± 0.04	1796 ± 71	95.44 ± 2.09	0.62 ± 0.34
*Verrucomicrobiae*	*Akkermansiaceae* (UBA985 sp003527555)	5	2.57 ± 0.79	48.44 ± 0.71	2410 ± 376	88.6 5 ± 18.1	1.13 ± 0.39
*Poseidoniia*	Ca. *Poseidoniaceae* (MGIIa-L1 sp.)	1	1.56	52.10	1772	61.82	1.60

^*^For species with more than one MAG, average values and standard deviations are shown

### Taxonomic affiliation of MAGs

**Fig. 1:**
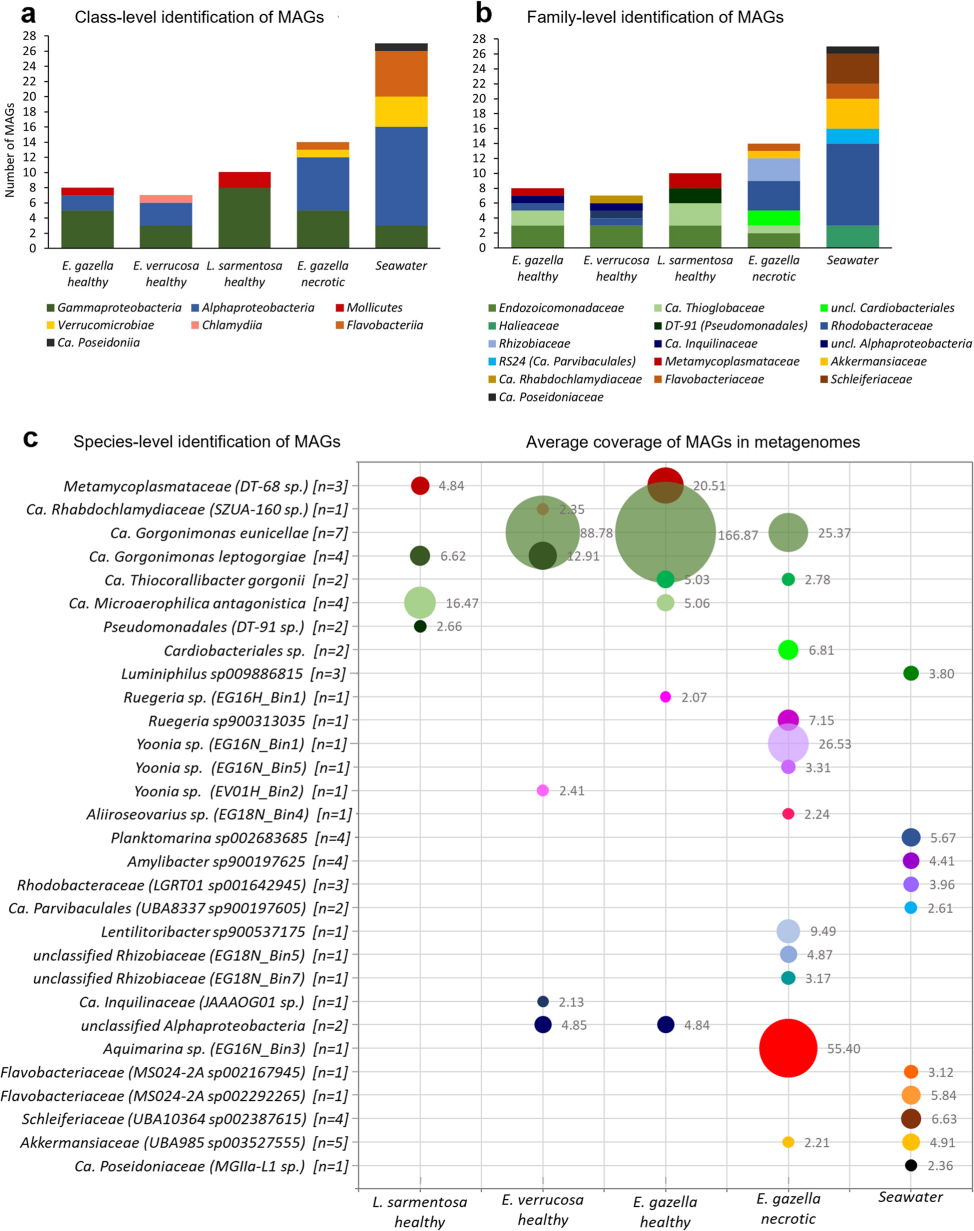
Taxonomic affiliation at **a** class, **b** family, and **c** species level of the 66 metagenome-assembled genomes (MAGs) obtained from the microbial metagenomes of healthy and necrotic *Eunicella gazella* tissue, healthy *E.* *verrucosa*, and *Leptogorgia* *sarmentosa*, and seawater. Average genome coverage values (inferred by mapping unassembled reads against MAG contigs) represent a relative abundance estimate of the prokaryotes in each microhabitat (**c**)

The 66 MAGs belonged to six phyla, seven classes, 15 orders, 16 families, and at least 30 species as defined by 95% ANI thresholds (Fig. 1a–c; Table S1, Additional file 2). The 21 *Gammaproteobacteria* MAGs retrieved from octocorals all represented so-far uncultured and unclassified lineages, with 11 MAGs affiliating with the *Endozoicomonadaceae* family (Fig. 1b). Of these, nine derived from the microbiomes of healthy octocoral tissue and two from necrotic tissue. Six octocoral-derived MAGs affiliated with the family Ca. *Thioglobaceae*, five of them obtained from the microbiomes of healthy tissue. Moreover, two *Gammaproteobacteria* MAGs from healthy *L.* *sarmentosa* were affiliated with candidate taxon DT-91 of the order *Pseudomonadales*, while two MAGs from necrotic *E.* *gazella* were identified as unclassified *Cardiobacterales*. Contrarily, the three *Gammaproteobacteria* MAGs found in seawater belonged to the genus *Luminiphilus* (*Halieaceae*, *Cellvibrionales*). Notably, four MAGs from healthy octocoral tissue were affiliated with intracellular bacterial symbionts [58] of the families *Metamycoplasmataceae* and Ca. *Rhabdochlamydiaceae*. The five *Alphaproteobacteria* MAGs from healthy octocoral tissue were either unclassified at order level (*N* = 2) or belonged to the candidate family *Inquilinaceae *and the *Rhodobacteraceae* genera *Ruegeria* and *Yoonia* (Fig. 1c). Among the MAGs obtained from necrotic *E.* *gazella* tissue was also one affiliating with the genus *Aquimarina* (*Bacteroidetes*), a taxon frequently cultured from octocorals [19, 28], as well as three MAGs affiliating with the *Rhizobiaceae* family, one of them identified as *Lentilitoribacter*. Overall, little overlap was observed at species level between MAGs reconstructed from healthy octocoral tissue versus necrotic tissue versus seawater. Of the 11 bacterial species recovered from healthy octocorals, only two species, the here proposed Ca. *Gorgonimonas* *eunicellae* and Ca. *Thiocorallibacter* *gorgonii*, were also recovered from necrotic samples. However, their average genome coverage, a proxy for relative abundance, was much higher in healthy than necrotic *E.* *gazella* tissue (nearly sevenfold difference in coverage for Ca. *G.* *eunicellae* and nearly twofold difference in coverage for Ca. *T.* *gorgonii* in healthy vs. necrotic samples; Fig. 1c).

### Phylogenomics of *Endozoicomonadaceae* and Ca. *Thioglobaceae* MAGs

**Fig. 2:**
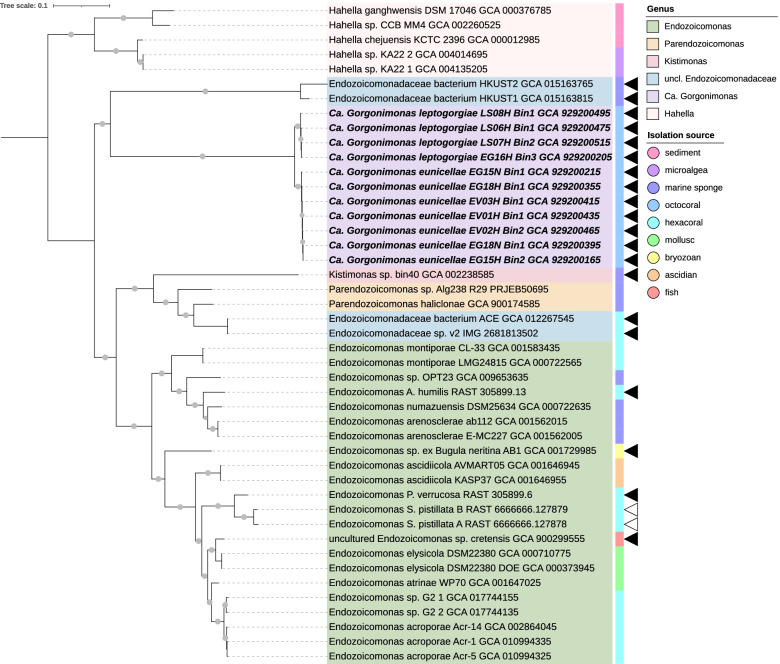
Phylogenomic analysis of the *Endozoicomonadaceae* family using the speciesTreeBuilder v.01.0. Evolutionary history was inferred by using a maximum likelihood method (FastTree 2) based on alignment similarity of a set of 49 cores, universal genes defined by Clusters of Orthologous Groups of proteins (COG) gene families. Gray dots on the branches indicate bootstrap support of > 70%. Black triangles indicate a MAG, white triangles a SAG, and the remaining genomes were from isolates. The 11 *Endozoicomonadaceae* MAGs of this study are highlighted in bold-italics. All other *Endozoicomonadaceae* genomes (*n* = 29) were publicly available on RAST, IMG, or NCBI. Assembly accession numbers are given next to the strain names. Five *Hahella* spp. genomes of the closely related *Hahellaceae* family were used as outgroup to root the tree. The colored bar next to the tree shows the isolation source of the genomes. Note that all *Endozoicomonadaceae* genomes (including MAGs and SAGs) are derived from the microbiomes of marine animals, mainly marine invertebrates. The tree is drawn to scale and was style edited in iTOL

Phylogenomics analysis of the *Endozoicomonadaceae* family comprised 29 publicly available type genomes, MAGs, and SAGs plus the 11 octocoral-derived MAGs retrieved in this study (Fig. 2). The latter formed their own, well supported and deeply branching clade, separate from the genomes of all formally described genera with cultured representatives (i.e., *Kistimonas*, *Parendozoicomonas*, and *Endozoicomonas*). This clade comprised two subclusters, each representing a novel species, sharing ~ 89.8% ANI between them. Subcluster 1 contained seven MAGs, all obtained from *Eunicella* hosts. Subcluster 2 comprised four MAGs, three from *L.* *sarmentosa* and one from *E.* *gazella*. The closest type strain genome to the 11 *Endozoicomonadaceae* MAGs was *Endozoicomonas* *atrinae* GCA_001647025T, which shared only 52–53% AAI with them (Table S1, Additional file 2), well below the 65% threshold considered by MiGA for same-genus classification. This indicates that the 11 *Endozoicomonadaceae* MAGs represent two distinct species, forming a novel yet uncultured genus unique to temperate octocorals. We propose the names Candidatus *Gorgonimonas* *eunicellae* (corresponding to subcluster 1) and Ca. *Gorgonimonas* *leptogorgiae* (corresponding to subcluster 2) for the two species.

Phylogenomics inference of the Ca. *Thioglobaceae* family showed that the six octocoral-derived *Thioglobaceae *MAGs formed two separate clusters, representing distinct, novel species as judged by ANI values way below 80% compared with the remainder genomes of the family (Fig. S1, Additional file 1). The first clade comprised two *E.* *gazella*-derived MAGs which formed a subcluster within other Ca. *Thioglobaceae* clusters of the genera *Thioglobus* and *Thiomultimodus*. The second clade was composed of the other four *Thioglobaceae* MAGs, derived from healthy *L.* *sarmentosa* and *E.* *gazella* tissue, which formed a well-supported, deeply branching phylogenomic node on their own, sharing only 46% AAI with genomes of their closest type strains, namely *Sulfurivirga* *caldicuralii* GCA_900141795T and *Thiohalobacter* *thiocyanaticus* GCA_003932505T (Table S1, Additional file 2). This indicates that the six octocoral-derived Ca. *Thioglobaceae* MAGs not only represent two distinct species but most likely two distinct, novel genera, here proposed Ca. *Thiocorallibacter* *gorgonii* and Ca. *Microaerophilica* *antagonistica* (Fig. S1, Additional file 1), which so far lack cultured representatives.

### Functional profiling of MAGs from octocoral and seawater microbiomes

**Fig. 3:**
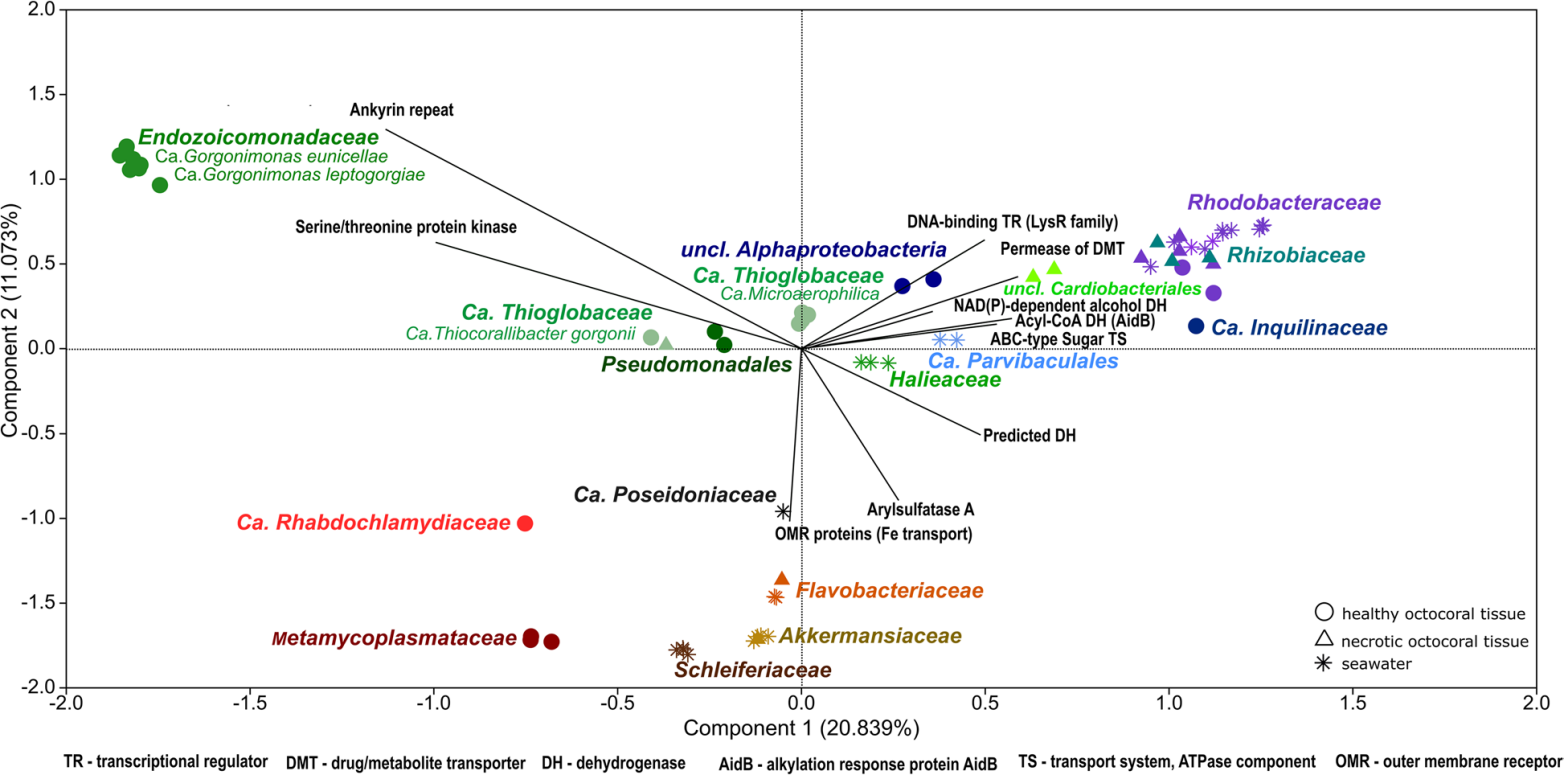
Multivariate analysis of COG profiles of the 66 MAGs of this study. Principal components analysis (PCA) was performed using the Euclidean distance matrix calculated from Hellinger-transformed abundance data. The ordination is shown in Eigenvalue scale. MAGs from healthy octocoral samples are represented by circles, MAGs from necrotic octocoral samples by triangles, and MAGs from seawater by asterisks. Symbols are colored according to the family-level, taxonomic affiliation of the MAGs with family names written next to them. Black arrows represent the top ten COG functions (i.e., COG0666, COG0515, COG3119, COG1629, COG1960, COG0673, COG0583, COG0697, COG1028, COG3839, in that order) which contributed most to the dissimilarities between these MAGs at order level, as revealed by a SIMPER test. Note the highly distinct clustering of the *Endozoicomonadaceae* MAGs, statistically supported by a one-way PERMANOVA test (*P* < 0.001) with 999 permutations and driven by the high copy number of genes encoding for ankyrin repeat proteins (COG0666) and serine/threonine protein kinases (COG0515). See also Tables S3 and S4 (Additional file 2) for complete COG profiles and SIMPER results

Multivariate analysis based on COG functional profiles showed that the MAGs grouped primarily according to their (order level) taxonomic affiliations (PERMANOVA, *F* = 9.869, *P* = 0.0001) (Fig. 3). The 11 *Endozoicomonadaceae* MAGs formed a very tight cluster, much distant from all other MAGs. Such separate clustering was mostly determined by the high copy number of ankyrin repeat motifs (COG0666) and serine/threonine protein kinase-encoding genes (COG0515) on the *Endozoicomonadaceae* MAGs. SIMPER analysis showed that these two COGs were indeed the functions that contributed most to the dissimilarity between all MAGs at order level (Tables S3, S4, Additional file 2).

The six Ca. *Thioglobaceae* MAGs formed two, well-separated clusters in the ordination space, one comprising the two Ca. *Thiocorallibacter* *gorgonii* MAGs and another one with the four Ca. *Microaerophilica* *antagonistica* MAGs, congruent with our phylogenomic assessment. The positioning of *Alphaproteobacteria *MAGs in the PCA diagram was influenced, among others, by the presence and abundance of genes encoding for LysR family transcriptional regulator (COG0583), ABC sugar transport system (COG3839), Acyl-CoA and NAD(P)-dependent alcohol dehydrogenases (COG1960 and COG1028), and drug metabolite transporters (COG0697).

### Functional features enriched in *Endozoicomonadaceae* symbionts of octocorals

**Fig. 4:**
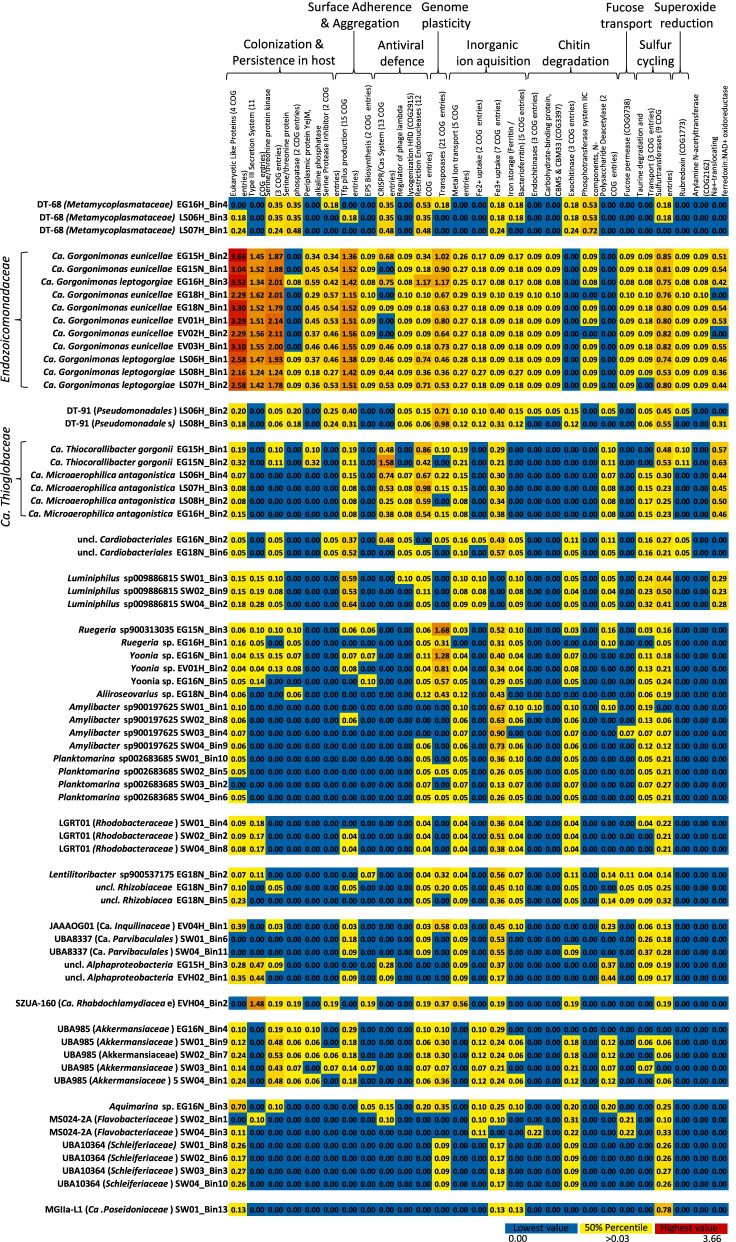
Functional analysis of the 66 MAGs according to their COG profiles. Values for each entry represent the percentage of COGs assigned to a given function relative to the total number of COGs annotated per MAG. The color code from blue over yellow to dark red reflects an increase in the percentage of COGs related to each function (blue = 0% (minimum value); yellow = 50% percentile; dark red = 3.66% (maximum value). When functions were represented by more than one COG entry across the data set, the coding sequence (CDS) counts of these functionally belonging COGs were summed, and the number of COG entries that contributed to each function is given in brackets behind the COG description. Identification of COGs that contributed to this figure can be found in Tables S5A and S5B, Additional file 2

**Fig. 5:**
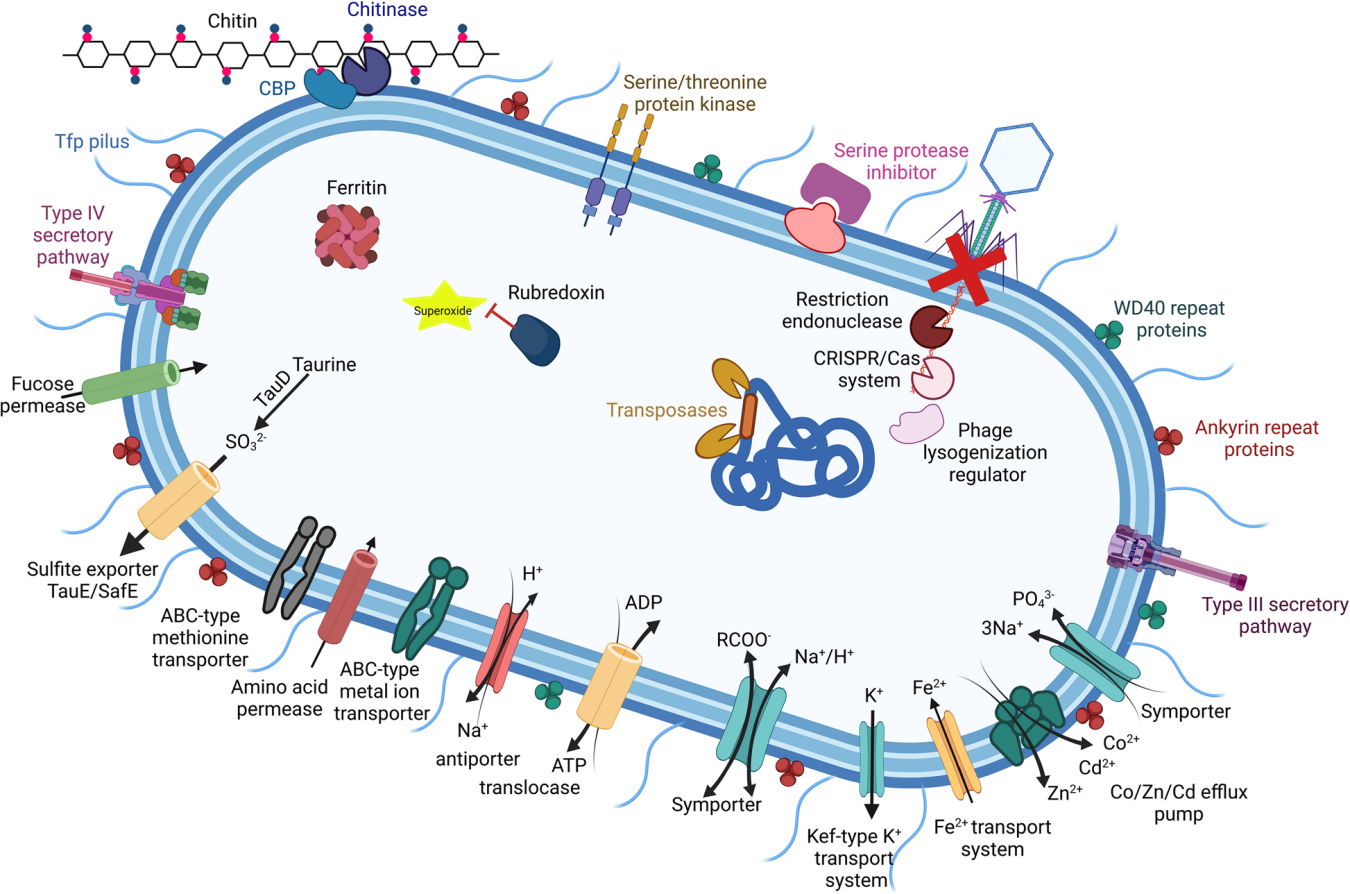
Schematic drawing of the predicted metabolic features (based on COG functional annotations) of the here described Ca. *Gorgonimonas* (*Endozoicomonadaceae*) symbionts of octocorals. Detailed information on the COG functions characteristic of the Ca. *Gorgonimonas* MAGs can be found in Fig. S2 of Supplementary file 1 and Tables S4 and S5 of Supplementary file 2. CBP, chitin-binding protein. The figure was created in BioRender.com

The 11 *Endozoicomonadaceae* MAGs were drastically enriched in COGs related to eukaryotic-like proteins (Fig. 4), mainly ankyrin repeats (*q* < 0.0001; Welch’s test) and, to a lesser extent, WD40 repeats and tetratricopeptide repeats (Table S5, Additional file 2, Fig. S2, Additional file 1). These MAGs also displayed high abundance of COG entries related to the type 3 secretion system (*q* < 0.0001; Welch’s test), serine/threonine protein kinases, serine protease inhibitors (*q* < 0.0001; Welch’s test), and the membrane-anchored periplasmic protein YejM. Other typical features of all 11 *Endozoicomonadaceae* MAGs were several COG entries associated with type 4-pilus (tfp) production (Figs. 4 and 5), and the consistent presence of COG3206 encoding a protein involved in exopolysaccharide (EPS) biosynthesis, which was not observed to such extent in any of the other 55 MAGs investigated here. The two *Endozoicomonadaceae* species identified in this study were distinguished by a consistent presence of serine/threonine phosphatase encoding genes on the four MAGs of Ca. *Gorgonimonas* *leptogorgiae* which were absent on the seven MAGs of Ca. *G.* *eunicellae.*

**Fig. 6:**
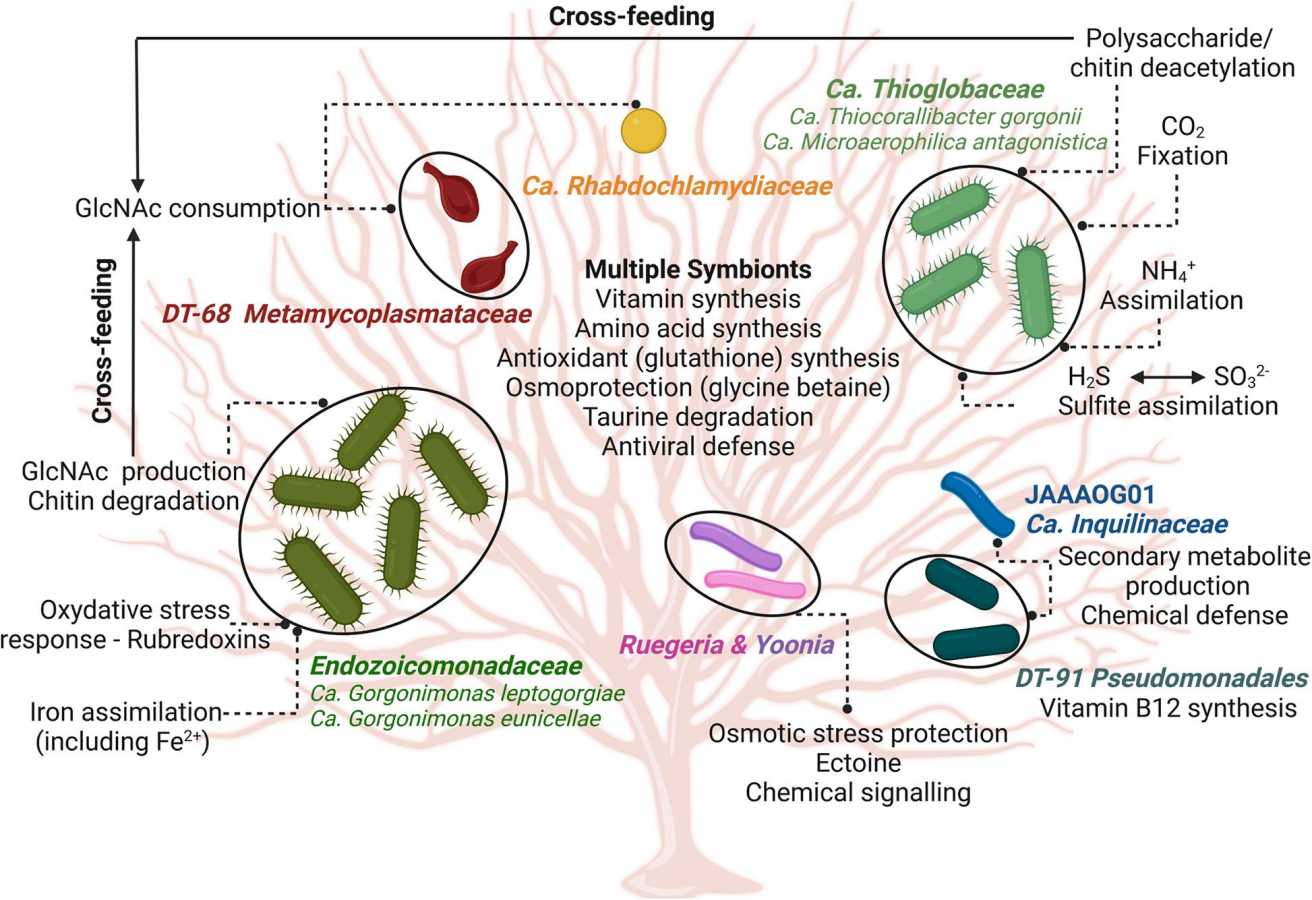
Presumed niche partitioning and metabolic interaction in bacterial symbionts of azooxanthellate octocorals. The schematic overview was inferred from the functions encoded by the MAGs of the 25 bacterial symbionts recovered from healthy *E.* *gazella*, *E.* *verrucosa*, and *L.* *sarmentosa* samples. Octocoral symbionts presumably participate in carbon, nitrogen, and sulfur cycling, amino acid, and B vitamin provision, chemical defense, and oxidative and osmotic stress protection. While *Endozoicomonadaceae* possess endo-chitinases to break down large chitin polymers; others, including *Metamycoplasmataceae* and Ca. *Thioglobaceae* MAGs, possess exo-chitinase and polysaccharide deacetylase genes, respectively, suggesting substrate cross-feeding in the octocoral holobiont. The figure was created in BioRender.com

The *Endozoicomonadaceae* MAGs show capacity for pyruvate metabolism and to convert acetyl-CoA to acetate via acetyl phosphate, through a phosphate acetyl transferase (EC 2.3.1.8) and an acetate kinase (EC 2.7.2.1), respectively, a process able to generate ATP independently from aerobic conditions (Fig. S3, Additional file 1). The consistent presence of pyruvate formate-lyase-activating enzyme encoding genes (COG1189) on the 11 *Endozoicomonadaceae* genomes (Fig. S2, Additional file 1, Welsh’s test *q* < 0.0001) further suggests that these symbionts may supply the citric acid cycle with acetyl-CoA from pyruvate during anaerobic glycolysis. Indication for adaptation of *Endozoicomonadaceae* symbionts to suboxic conditions was also found through the consistent presence of feoA/B genes (COG1918, COG0370) encoding for ferrous iron (Fe^2+^) uptake systems, distinguishing this taxon from the other 55 MAGs of this study (Welch’s *t*-test, *q* < 0.0001). In this regard, *Endozoicomonadaceae* MAGs were also distinguished by the presence of rubredoxin encoding genes (Figs. 4, 5 and 6; Fig. S2, Additional file 1), a class of iron-containing proteins which play an important role in superoxide reduction that can be found in several anaerobic and sulfate-reducing bacteria.

All *Endozoicomonadaceae* MAGs possessed a gene encoding an endo-chitinase (COG3469) involved in the extracellular breakdown of chitin polymers (Figs. 4, 5 and 6). We found a high degree of novelty within these 11 endo-chitinase encoding genes, as they clustered into two distinct groups and possessed less than 50% amino acid sequence similarity to publicly available endo-chitinases (Blastp search). Remarkably, these groups mirror the phylogenomic relatedness of the *Endozoicomonadaceae* MAGs, representing two endo-chitinase gene clades, each from Ca. species *Gorgonimonas* *leptogorgiae* and *G.* *eunicellae* (Fig. S4, Additional file 1). Protein family (Pfam) analysis confirmed the presence of a GH18 domain with an active site on all 11 genes. These genes were all complete (start and stop codon present) and carried a signal peptide sequence, indicating the protein can be excreted from the cell (Table S6A, Additional file 2). We also screened all publicly available genomes from cultured and uncultured *Endozoicomonadaceae* representatives and detected endo-chitinases on 32 out of 42 *Endozoicomonadaceae* genomes (Table S6B, Additional file 2). Moreover, genes for exo-chitinases, the enzymes that cleave the smaller chito-oligomer products into mono-sugars, were present in *Metamycoplasmataceae*, Ca. *Rhabdochlamydiaceae*, and *Pseudomonadales* symbionts of octocorals, while polysaccharide/chitin deacetylase encoding genes, which lead to the production of chitosan, were found in Ca. *Thioglobaceae* and several *Alphaproteobacteria* symbionts.

### Metabolic inference of Ca. *Thioglobaceae *symbionts of octocorals

The Ca. *Thioglobaceae* MAGs were significantly enriched in CRISPR/Cas system-associated endoribonuclease Cas2 (COG1343) (Fig. S5, Additional file 1). Several other CRISPR/Cas protein-encoding genes were found on all Ca. *Thioglobaceae*, all *Metamycoplasmataceae*, and many *Endozoicomonadaceae* MAGs (Fig. 4; Table S5, Additional file 2). Ca. *Thioglobaceae* MAGs were further significantly enriched in Na^+^-translocating ferredoxin:NAD^+^ oxidoreductase (Rnf complex) encoding genes (Fig. S5, Additional file 1), which were also present in great abundance in nine of the 11 *Endozoicomonadaceae* MAGs. An assimilatory sulfite reductase (EC 1.8.1.2) was also found on the Ca. *Thioglobaceae* MAGs, pointing towards a role in sulfur cycling in the octocoral holobiont. The four Ca. *Microaerophilica* *antagonistica* MAGs encoded for versatile taurine utilization pathways and its metabolization to aminoacetaldehyde and sulfite, via taurine deoxygenase (TauD; EC 1.14.11.17), or to sulfoacetaldehyde via taurine-pyruvate-aminotransferase (EC 2.6.1.77) (Fig. S6, Additional file 1).

All Ca. *Thioglobaceae* MAGs showed an extensive genetic repertoire for ammonium assimilation and transformation of inorganic nitrogen into amino acids, possessing genes coding for glutamine and asparagine synthetase (EC 6.3.1.2; EC 6.3.5.4), L-asparaginase (EC 3.5.1.1), glutamine amidotransferase chain of NAD synthetase (EC 6.3.1.5), aminoethyltransferase (EC 2.1.2.10), and glutamate synthase (EC 1.4.1.13; EC 1.4.7.1). The latter was significantly enriched in this symbiotic family compared to the other 60 MAGs obtained in this study (one-sided Welch’s test, *p* > 0.001, Fig. S5, Additional file 1). Finally, Ca. *Thiocorallibacter* *gorgonii* MAG EG15H_bin1 stood out as the only MAG harboring ribulose 1,5-bisphosphate carboxylase — RuBisCo (EC 4.1.1.39, COG4451, COG1850) and several other genes involved in the reductive dicarboxylate cycle (Fig. S7, Additional file 1), suggesting a chemoautotrophic lifestyle and “dark carbon fixation” ability of this octocoral symbiont.

### Genomic features of prokaryotes from necrotic octocoral tissue and surrounding seawater

Alongside several *Alphaproteobacteria* MAGs of the *Rhodobacteraceae* and *Rhizobiaceae* families, we recovered an *Aquimarina* sp. (*Flavobacteriaceae,* *Bacteroidetes*) MAG with high genome coverage, suggesting high abundance of this taxon in necrotic *E.* *gazella* tissue. The *Aquimarina* MAG not only harbored a variety of SM-BGCs typical for the genus [59, 60] but also a high number of eukaryotic-like proteins, particularly leucine-rich and tetratricopeptide repeats, likely facilitating its association with eukaryotic hosts. This MAG further harbored genes involved in chitin deacetylation and chitin oligomer utilization as well as several transposase encoding genes (Fig. 4), suggesting an ability of quick genome rearrangement to adapt to different environmental settings. However, the *Aquimarin*a MAG distinguished itself from the dominant symbionts obtained from healthy octocoral tissue by the presence of a virulence factor (COG2996) and nitric oxide reductase and arginase genes, indicating this species is equipped to attack the host’s immune system and to display opportunistic-to-pathogenic behavior, as discussed elsewhere [15, 60]. Underpinning their free-living lifestyle, the *Rhodobacteraceae* MAGs recovered from seawater are distinguished by the absence of serine-threonine protein kinases and serine protease inhibitors, and lack of the CRISPR/CAS system which we found enriched in many of the *Gammaproteobacteria *symbionts recovered from healthy octocorals. Likewise, the archaeal (Ca. *Poseidoniaceae*) MAG from surrounding seawater also lacked the aforementioned genes and other typical symbiosis features that would indicate host preference (Fig. 4), consistent with the notion of Ca. *Poseidoniaceae* spp. as primarily planktonic archaea [61]. It harbored, however, genes involved in the biosynthesis of four (i.e., B2, B3, B7, B9) of eight B vitamins and of some, mostly “nonessential” amino acids, e.g., alanine, serine, and glycine.

### B-vitamin and amino acid biosynthesis capacities in octocoral symbionts

All *Gammaproteobacteria *species obtained from healthy octocoral tissue possessed genes for the synthesis of at least six of eight B vitamins. For example, the *Endozoicomonadaceae* MAGs harbored genes for vitamin B1 (thiamine), B2 (riboflavin), B3 (niacin/NAD), B6 (pyridoxine), B7 (biotin), and B9 (folate) biosynthesis but lacked genes for B5 (pantothenic acid/CoA) and B12 (cobalamin) synthesis. The *Pseudomonadales* MAGs from healthy octocoral, however, possessed genes for the synthesis of all eight B vitamins (Table S7, Additional file 2). On the contrary, the *Metamycoplasmataceae* and *Rhabdochlamydiaceae* symbionts completely lacked B vitamin biosynthesis capacities, indicating genome streamlining congruent with their very small genome sizes. Moreover, both taxa possessed very limited coding potential for amino acid biosynthesis, harboring genes only for the synthesis of two and five of the 20 most common proteinogenic amino acids, respectively. The *Gammaproteobacteria* species from healthy octocorals, however, collectively harbored genes for the synthesis of 19 out of 20 proteinogenic amino acids, among them all nine “essential” amino acids, including the energetically costly methionine, histidine, and tryptophan (Table S7, Additional file 2). While the *Gammaproteobacteria* symbionts lacked the capacity to synthesize arginine, we identified arginine biosynthesis genes in an unclassified *Alphaproteobacteria* symbiont retrieved from healthy octocoral tissue. The *Alphaproteobacteria* species found in healthy octocorals were in general also well equipped to synthesize most B vitamins, including B12, and “essential” amino acids.

### Secondary metabolite biosynthetic potential of octocoral symbionts

**Fig. 7:**
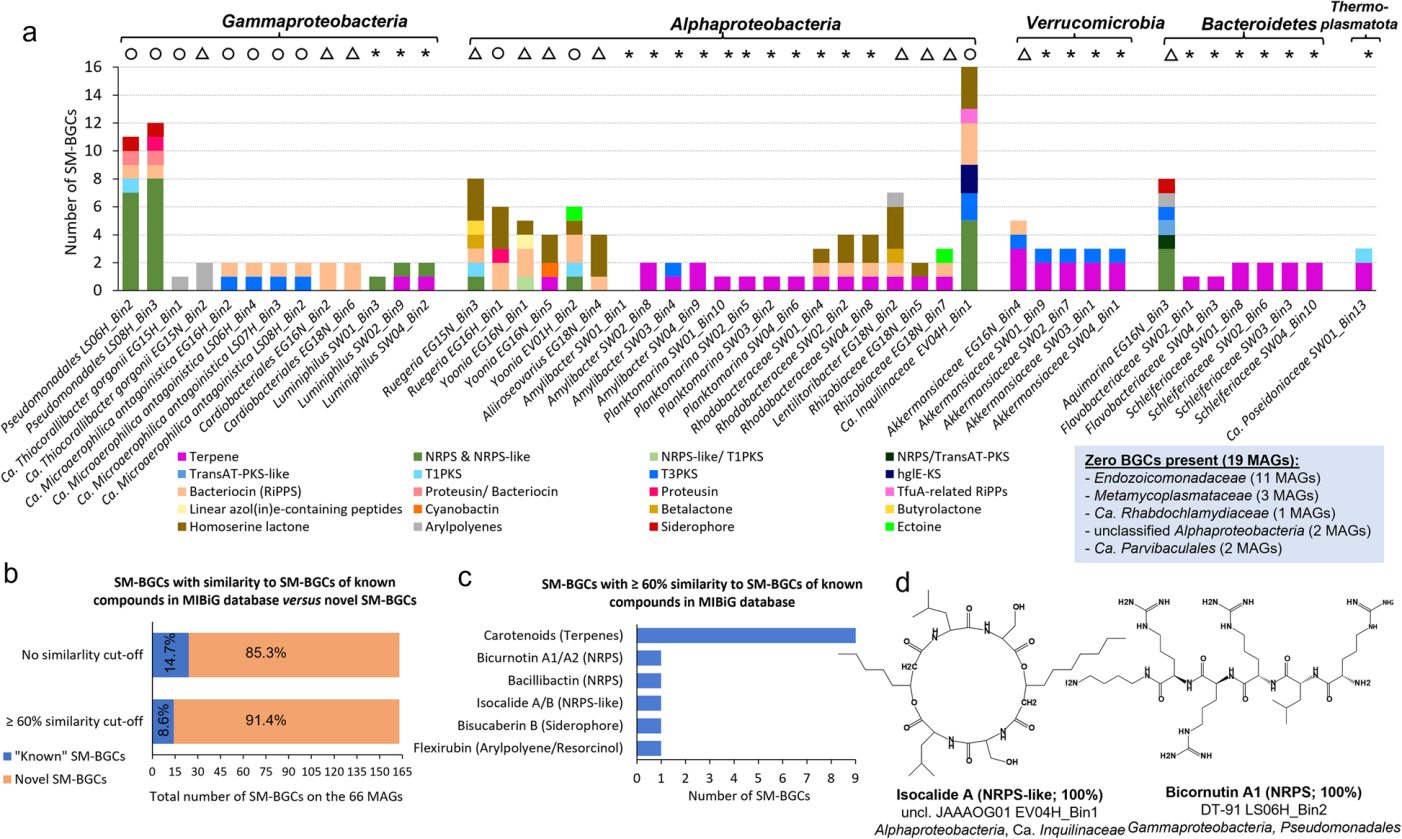
Secondary metabolite coding potential in each MAG obtained from microbial metagenomes of healthy and necrotic octocoral samples and seawater. **a** Distribution of secondary metabolite biosynthesis gene clusters (SM-BGCs) across the 66 MAGs of this study. Symbols above bars indicate the origin of each MAG (same as in Fig. 3). SM-BGC counts per compound class were obtained using antiSMASH v.5.0 with default detection strictness (and all extra features on). PKS, polyketide synthase; hglEKS, heterocyst glycolipid synthase-like PKS; NRPS, nonribosomal peptide synthetase cluster; TfuA related, TfuA-related ribosomal peptides. **b** Percentage of SM-BGCs, identified on the 66 MAGs, with a hit to SM-BGCs of known compounds present in the MIBiG database. Only 14 of the 163 SM-BGCs obtained in this study shared 60% or more similarity to SM-BGCs of known compounds, highlighting the high degree of novelty encoded in these MAGs. **c** Predicted compounds among the SM-BGCs identified in the MAGs. **d** Chemical structures of isocalide A and bicornutin A1, encoded in the octocoral-derived MAGs of JAAAOG01 EV04H_Bin1 (Ca. *Inquilinaceae*, *Alphaproteobacteria*) and DT-91 LS06H_Bin2 (*Pseudomonadales*, *Gammaproteobacteria*). Structures were drawn with ChemDraw v. 12.0.2

Genome mining with antiSMASH revealed that 46 of 66 MAGs analyzed in this study harbored between one and 16 (Ca. *Inquilinaceae* EV04_Bin1) SM-BGCs, while 20 MAGs lacked SM-BGCs, among them all *Endozoicomonadaceae*, *Metamycoplasmataceae*, Ca. *Rhabdochlamydiaceae*, and unclassified *Alphaproteobacteria* MAGs from octocorals (Fig. 7a; Table S8, Additional file 2). Notably, the two Ca. *Thioglobaceae* species presented distinct secondary metabolite coding potential. While Ca. *Thiocorallibacter* *gorgonii* MAGs harbored one or two arylpolyene cluster(s) (which may function as antioxidants), Ca. *Microaerophilica* *antagonistica* MAGs harbored one T3PKS and one bacteriocin/RiPP SM-BGC each, which may indicate antagonistic potential. The two *Pseudomonadales* MAGs from healthy *L.* *sarmentosa* samples showed rich SM-BGC profiles, with 7–8 NRPS and 2–3 bacteriocin/RiPP/proteusin clusters, plus a siderophore SM-BGC (Figs. 6 and 7a).

Only 24 out of 163 SM-BGCs detected across all MAGs showed some homology with SM-BGCs encoding known compounds present in the MIBiG database, with 14 SM-BGCs sharing a similarity of 60–100% (Fig. 7b; Table S9, Additional file 2). One of the NRPS clusters of *Pseudomonadales* MAG LS06H_Bin2 showed 100% similarity to the NRPS cluster of the antimicrobial peptide bicornutin A [62] (Fig. 7c and d). Another NRPS cluster with 100% similarity to a SM-BGC of a known compound, namely the antibiotic and cyclic depsipeptide isocalide A [63], was identified on the Ca. *Inquilinaceae* MAG from healthy *E.* *verrucosa*, which was also the MAG with the richest SM-BGC profile across the entire dataset*.*

## Discussion

This is the first functional genomics study of uncultivated symbionts of octocorals. Notably, MAGs of dominant *Endozoicomonadaceae *symbionts were retrieved from all octocoral species and from nine out of 10 healthy coral specimens analyzed, strengthening the current understanding of these symbionts as indicators of coral health. Extensive amino acid and B vitamin biosynthesis capacities were common traits of the dominant *Gammaproteobacteria* fraction (*Endozoicomonadaceae*, Ca. *Thioglobaceae*, and *Pseudomonadales* MAGs) characteristic of healthy octocorals (summarized in Fig. 6). This outcome corroborates recent research on scleractinian coral symbionts, which suggested amino acid and vitamin biosynthesis among the key traits for holobiont functioning [23, 26]. Our metagenomic data suggest that cross-feeding and niche partitioning, particularly of chitin and its breakdown products, B vitamins, and amino acids, may be mechanisms to promote coexistence and energy conservation among symbionts of azooxanthellate octocorals, which deserves further investigation and experimental validation in the future. Niche partitioning is proposed by sharp differences in secondary metabolite biosynthesis potential between symbiotic lineages, whereby *Endozoicomonadaceae*, *Metamycoplasmataceae* and *Chlamydiales* symbionts did not possess a single SM-BGC, indicating they might not engage in chemical defense. Instead, *Pseudomonadales* and specific *Alphaproteobacteria* symbionts of healthy octocorals are likely to fulfill this task. They possessed the richest secondary metabolism across all 66 MAGs examined, including the genomic blueprint for the biosynthesis of antibiotics such as isocalide A [63]. Below, we highlight key genomic features and putative mechanisms of interaction among octocoral symbionts, focusing on the *Endozoicomonadaceae*-*Thioglobaceae*-*Metamycoplasmataceae* triad.

### Discovery of novel *Endozoicomonadaceae* symbionts highly adapted to life in octocorals

This study uncovered two species of a novel *Endozoicomonadaceae* genus so far unique to temperate octocorals, here denoted Ca. *Gorgonimonas* *eunicellae* and Ca. *Gorgonimonas* *leptogorgiae*. These *Gorgonimonas* MAGs showed many traits that facilitate colonization of and persistence inside the host animal, including high abundances of ankyrin repeats. Studies on marine sponges showed that bacteria expressing ankyrin genes avoid phagocytosis by sponge amoebocytes, thus residing inside the sponge by evading its immune system [64]. A similar mechanism likely exists in coral-associated bacteria. Some bacteriophages also carry genes for ankyrin biosynthesis and possibly even transfer this information across different microbial symbionts [65]. Our earlier research demonstrated that ankyrin repeats are enriched in the microbial metagenomes of healthy octocorals [15], and this study now attributes this trait to specific *Endozoicomonadaceae* symbionts. A high copy number of ankyrin repeats was recently found among some cultured *Endozoicomonas* strains [21, 26]. Moreover, *Endozoicomonas* *marisrubri* 6c was shown to upregulate ankyrin repeat expression upon exposure to scleractinian coral extract [26]. This study is the first to show the enrichment of this trait in dominant, deeply branching, and uncultivated *Endozoicomonadaceae* lineages within the octocoral microbiome, further underpinning the importance of ankyrin repeats in coral symbioses. *Gorgonimonas* MAGs also possessed a high abundance of genes related to the type 3 secretion system, serine/threonine protein kinases, serine protease inhibitors, and the protein YejM. These features enable colonization and interference with host defense mechanisms among bacterial pathogens [66–68]. There is, however, growing evidence that beneficial bacteria use similar mechanisms to suppress immune responses and facilitate host colonization [69]. Indeed, parasitic and mutualistic symbioses often share similar evolutionary histories. Thus, host-microbe interactions may rather represent a flexible gradient, known as the “parasite-mutualist continuum,” than a static binary system, and can shift and evolve depending on ecological context [70]. To accurately discern the beneficial-to-parasitic behavior of coral symbionts in a context-dependent manner, future studies need to combine multi-omics analyses with controlled in vivo mesocosm experiments.

All *Gorgonimonas* MAGs showed great capacities to produce tfp pili, bacterial surface appendages required for adherence to solid surfaces or cells, including host cells [71]. Tfp pili are also involved in bacterial aggregation (agglutination) and flagellum-independent movement on solid surfaces via gliding or twitching motility, coupled with chemotaxis [71]. CARD-FISH analysis demonstrated that *Endozoicomonas* bacteria indeed aggregate in dense, cyst-like structures inside the coral endoderm [72]. The here identified tfp pili likely provide the mechanisms for *Endozoicomonadaceae* symbionts to form such aggregations and attach to and move inside their host.

### Ca. *Thioglobaceae* genomes code for a sophisticated antiviral defense system and chemoautotrophic metabolism

The Ca. *Thioglobaceae* MAGs were significantly enriched in CRISPR/Cas protein-associated genes, also found in all *Metamycoplasmataceae* (DT-68) and many *Endozoicomonadaceae* MAGs. These proteins are prokaryotic defense mechanisms against bacterial viruses and foreign DNA known to be enriched in the microbiomes of marine sponges [65, 73] and octocorals [15]. A recent study of the *Thioglobaceae* pan-genome showed that CRISPR/Cas systems are relatively enriched in mussel, sponge, and scleractinian coral symbionts compared to free-living *Thioglobaceae* lineages [74]. The Ca. *Thioglobaceae* and *Endozoicomonadaceae* MAGs of this study exhibited intraspecific variability in CRISPR/Cas gene numbers, leading to a flexible pan-immune system within host-associated populations, as suggested elsewhere [75].

We found one Ca. *Thioglobaceae* MAG to harbor RuBisCo and other genes for light-independent carbon fixation and chemoautotrophic lifestyle — as known for some *Thioglobaceae* *s*ymbionts from deep-sea sponges and hydrothermal vent bivalves [76]. It could represent an alternative carbon fixation route in azooxanthellate octocorals that lack photosynthetic Symbiodinaceae and other algae symbionts. It also indicates that the ecological range of chemoautotrophic, symbiotic *Thioglobaceae* extends beyond hydrothermal vent and deep-sea ecosystems well into mesophotic zones and above.

Beyond their putative role in carbon supply, most Ca. *Thioglobaceae* MAGs possessed the genetic repertoire for ammonium assimilation, sulfur cycling, and taurine processing within the octocoral holobiont. Taurine is a sulfur-containing amino acid widely found in animal tissue, and several studies suggested a role of sponge and coral symbionts in sulfur cycling via taurine metabolism [21, 23, 73, 77]. However, as observed in this study, taurine utilization is a widespread trait among marine host-associated and free-living, seawater-derived *Alpha-* and *Gammaproteobacteria* and not restricted to obligate symbiotic clades.

### *Endozoicomonadaceae*, Ca. *Thioglobaceae*, and *Metamycoplasmataceae* symbionts may adopt a facultative anaerobic lifestyle

We found genome-based evidence for *Endozoicomonadaceae*, Ca. *Thioglobaceae*, and *Metamycoplasmataceae* symbionts to possess a facultative anaerobic metabolism and the ability to thrive in low oxygen conditions. *Endozoicomonadaceae* symbionts may generate energy without oxygen via the pyruvate metabolism and with the help of acetate kinases and pyruvate formate lyases [78, 79]. All Ca. *Thioglobaceae* and most *Endozoicomonadaceae* MAGs also harbored Na^+^-translocating ferredoxin:NAD^+^ oxidoreductase (Rnf complex) encoding genes. The Rnf complex is involved in anaerobic respiration and alternative routes of energy generation by translocating sodium across the cell membrane, a common feature of anaerobic (acetate forming) acetogens (e.g., *Acetobacterium* *woodii*) [80]. Recent studies demonstrated that the Rnf complex is not limited to acetogens and instead can be found in several Gram-negative, facultative anaerobic bacteria [81–83]. The *Metamycoplasmataceae *(DT-68) and some Ca. *Thioglobaceae* MAGs further possessed lactate dehydrogenase encoding genes to ferment pyruvate into lactate and thus recycle NADH + H^+^, for glycolysis to continue under anaerobic conditions*.* Facultative anaerobe symbionts and anaerobic metabolism seem to play a crucial role in nutrient cycling in marine sponges [84]. Few cultured *Endozoicomonas* strains from octocorals are classified as facultative anaerobes, such as type strains *Endozoicomonas* *euniceicola* and *E.* *gorgoniicola* [22], indicating that octocoral holobionts might benefit from the facultative anaerobic lifestyles of their symbionts. The consistent presence of genes coding for ferrous iron (Fe^2+^) uptake systems on all *Endozoicomonadaceae* MAGs provides further support for this hypothesis, since Fe^2+^ is usually available in anaerobic environments while ferric iron (Fe^3+^) is dominant under oxygenated conditions. Organisms frequently exposed to oxygen limitation in their natural habitat rely on ferrous iron uptake mechanisms [85]. The 11 *Endozoicomonadaceae *MAGs were also enriched in ferritin encoding genes for iron storage, likely supporting growth of these symbionts during iron starvation [86], a situation they may frequently encounter in the host animal. Iron is indeed a limiting micronutrient in some marine environments and was shown to restrain primary productivity in corals [87, 88]. Together, such metabolic capacities could enable these symbionts not to drain their obligate aerobic host from oxygen but to provide it with iron whenever environmental oxygen is limited. Another distinguishing feature of the *Endozoicomonadaceae* symbionts of this study was the presence of rubredoxins, a class of iron-containing proteins found in several anaerobic and sulfate-reducing bacteria. Rubredoxins act as electron carriers in many biochemical processes and can play a crucial role in reducing reactive oxygen species [89–91]. Both effective oxidative stress response and iron-sequestration mechanisms were recently proposed as key beneficial traits of coral probiotics [92].

### Chitin breakdown and cross-feeding possibilities in the octocoral holobiont revealed

The distribution of endo-chitinase (*Endozoicomonadaceae*), exo-chitinase (*Metamycoplasmataceae* and several other symbiont taxa), and chitin deacetylase (Ca. *Thioglobaceae* and *Alphaproteobacteria* spp.) encoding genes across octocoral symbionts points towards their likely role in chitin processing and carbon and nitrogen turnover in their host. This indicates possible mechanisms of substrate cross-feeding [93] between symbiotic partners. Cross-feeding cascades among microbes may promote the evolution of small genomes as here observed, for example, in *Metamycoplasmataceae* (0.63 Mb) and Ca. *Rhabdochlamydiaceae* (1 Mb) symbionts. These symbionts may have evolved as auxotrophs, relying on the acquisition of certain nutrients and vitamins from other microbial partners of the coral holobiont. This may also reduce competition and enable coexistence of multiple host-associated taxa through functional specialization. Exo-chitinase (EC 3.2.1.52) activity was measured earlier in crude extracts of the octocoral *Gorgonia* *ventalina* [94]. Moreover, chitinolytic activity was reported in seven scleractinian coral species, and chitinase-like genes were identified in the genome of *Acropora* *digitifera* [95]. Raimundo and colleagues reported that the abundance of endo-chitinase and chitin-binding-protein encoding genes in healthy octocoral tissue levels up with those from surrounding environments [96]. Taken together, these observations suggest that chitinases are widely distributed among corals, many of which feed on chitin-rich phyto- and zooplankton [6]. These enzymes may also play a role in the animals’ defense against fungal infections [7], whereby chitinases from bacterial symbionts may further strengthen the coral immune system.

## Conclusion

This study examines the first microbial MAGs ever retrieved from octocorals, revealing that uncultured *Endozoicomonadaceae*, Ca. *Thioglobaceae*, and *Mycoplasmoidales* symbionts are seemingly well adapted to life in low oxygen conditions. Moreover, we identified a thus-far unanticipated, global role for *Endozoicomonadaceae* symbionts of corals in chitin processing and C and N cycling across benthic ecosystems. Other symbionts such as Ca. *Thioglobaceae* were well equipped to control bacteriophage attacks and to adopt a chemoautotrophic lifestyle. We conclude that the prokaryotic symbiome of octocorals participates in carbon (particularly chitin), nitrogen and sulfur cycling, amino acid and B vitamin provision, chemical defense, and oxidative and osmotic stress protection. Traits are not shared equally among all members of the symbiotic community; instead, niche partitioning and metabolic specialization among taxonomically unique symbionts may contribute to efficient functioning of and co-existence in the healthy octocoral holobiont.

## Supplementary Information

**Additional file 1: Figure S1.** Phylogenomic analysis of the Ca. *Thioglobaceae* family using SpeciesTreeBuilder v.01.0. **Figure S2.** COG functions (*N*=76) significantly enriched (*q*-value < 0.05) in the octocoral-derived *Ca. Gorgonimonas* (*Endozoicomonadaceae*) MAGs (*N*= 11, green), compared with all other MAGs (*N*=55) of this dataset. **Figure S3.** KEGG metabolic pathway map of the Ca. *Gorgonimonas* (*Endozoicomonadaceae*) MAGs of this study. **Figure S4.** Phylogeny of 101 full-length, bacterial endo-chitinase (EC 3.2.1.14) encoding genes, including the 11 endo-chitinase (GH18-family) genes from *Endozoicomonadaceae* MAGs (grey shadings) of this study. **Figure S5.** COG functions (*N*=34) significantly enriched (*p*-value < 0.05) in the Ca. *Thioglobaceae* MAGs (*N*= 6, dark green), compared with all other MAGs (*N*=60) of this dataset. An one-sided Welch’s t-test for unequal variances was performed in STAMP v.2.1.3. Multiple test correction was performed using the Benjamini-Hochberg correction (FDR). To further limit the displayed number of significant entries, an effect size filter was also applied, setting the “ratio of proportions” to 5.00. **Figure S6.** KEGG metabolic pathway map of the Ca. *Thioglobaceae* MAGs of this study. The metabolic map features glycolysis, pyruvate, nitrogen, sulfur, and taurine metabolism. EC numbers of enzymes catalyzing the reactions are given in rectangular boxes. EC numbers highlighted in green represent enzymes encoded on Ca. *Thioglobaceae* MAGs. Beige boxes indicate connections to other metabolic pathways active in these MAGs. **Figure S7.** KEGG carbon fixation map of Ca. *Thiocorallibacter* *gorgonii* MAG EG15H_Bin1 (Ca. *Thioglobaceae*). EC numbers of enzymes catalyzing the reactions are given in rectangular boxes. EC numbers highlighted in green represent enzymes encoded on this MAG. Beige boxes indicate connections to other metabolic pathways active in these MAGs. Orange dots highlight the substrates (Ribulose-1,5-bisphosphate, CO_2_, and H_2_O) and product (2 Glycerate-3-phosphate, 2 H^+^) of Rubisco (EC 4.1.1.39).

**Additional file 2: Table S1.** General genomic features, taxonomic classification and genome assembly accession numbers of each of the 66 metagenome-assembled genomes (MAGs) obtained from octocoral- and seawater-derived microbial metagenomes. **Table S2.** Overview of the number and quality of MAGs obtained from octocoral- and seawater-derived microbial metagenome samples. **Table S3.** Clusters of Orthologous Groups of proteins (COGs) annotation of the 66 MAGs analysed in this study. **Table S4.** Results of the SIMPER test performed on COG profiles (Hellinger-transformed abundances; Euclidean distances) of the 66 MAGs grouped at order level. **Table S5A.** Absolute abundances (counts) of COG functions shown in Figure 4 that distinguished the 66 MAGs the most (based on SIMPER and Welch's tests). **Table S5B.** Relative abundances of COG functions shown in Figure 4 that distinguished the 66 MAGs the most (based on SIMPER and Welch's tests). **Table S6A.** Features of the endo-chitinase (EC 3.2.1.14) genes found on the 11 *Endozoicomonadaceae *MAGs of this study. **Table S6B.** Genes involved in chitin degradation present on the 11 *Endozoicomonadaceae* MAGs of this study and other, publicly available *Endozoicomonadaceae* genomes. **Table S7.** Amino acid (n= 20) and B vitamin (n = 8) biosynthesis capacities (based on genomic evidence) of the 11 bacterial species recovered from the microbiomes of healthy octocoral tissue. **Table S8.** Secondary metabolite biosynthetic gene clusters (SM-BGCs) present on the 66 MAGs, annotated using antiSMASH bacterial version 5.0. **Table S9.** List of SM-BGCs with some level of homology to MIBiG database entries.

## Data Availability

The 66 MAGs are available in the European Nucleotide Archive (ENA) under the study accession number PRJEB50578 and sample accession numbers ERS10420767 — ERS10420805 (from octocorals) and ERS10422230 — ERS10422256 (from seawater). Assembly accession numbers for each MAG can be found in Table S1 of Additional file 2. All raw metagenome data are deposited under the study accession number PRJEB13222.
