# Supplementary material for: Metagenomics-resolved genomics provides novel insights into chitin turnover, metabolic specialization, and niche partitioning in the octocoral microbiome
Source: Microbiome. 2022 Sep 22;10:151. doi: 10.1186/s40168-022-01343-7 (PMC9502895; doi:10.1186/s40168-022-01343-7)
Supplement: Supplementary file 2 — Additional file 1: Figure S1. Phylogenomic analysis of the Ca. Thioglobaceae family using SpeciesTreeBuilder v.01.0. Figure S2. COG functions (N=76) significantly enriched (q-value < 0.05) in the octocoral-derived Ca. Gorgonimonas (Endozoicomonadaceae) MAGs (N= 11, green), compared with all other MAGs (N=55) of this dataset. Figure S3. KEGG metabolic pathway map of the Ca. Gorgonimonas (Endozoicomonadaceae) MAGs of this study. Figure S4. Phylogeny of 101 full-length, bacterial endo-chitinase (EC 3.2.1.14) encoding genes, including the 11 endo-chitinase (GH18-family) genes from Endozoicomonadaceae MAGs (grey shadings) of this study. Figure S5. COG functions (N=34) significantly enriched (p-value < 0.05) in the Ca. Thioglobaceae MAGs (N= 6, dark green), compared with all other MAGs (N=60) of this dataset. An one-sided Welch’s t-test for unequal variances was performed in STAMP v.2.1.3. Multiple test correction was performed using the Benjamini-Hochberg correction (FDR). To further limit the displayed number of significant entries, an effect size filter was also applied, setting the “ratio of proportions” to 5.00. Figure S6. KEGG metabolic pathway map of the Ca. Thioglobaceae MAGs of this study. The metabolic map features glycolysis, pyruvate, nitrogen, sulfur, and taurine metabolism. EC numbers of enzymes catalyzing the reactions are given in rectangular boxes. EC numbers highlighted in green represent enzymes encoded on Ca. Thioglobaceae MAGs. Beige boxes indicate connections to other metabolic pathways active in these MAGs. Figure S7. KEGG carbon fixation map of Ca. Thiocorallibacter gorgonii MAG EG15H_Bin1 (Ca. Thioglobaceae). EC numbers of enzymes catalyzing the reactions are given in rectangular boxes. EC numbers highlighted in green represent enzymes encoded on this MAG. Beige boxes indicate connections to other metabolic pathways active in these MAGs. Orange dots highlight the substrates (Ribulose-1,5-bisphosphate, CO2, and H2O) and product (2 Glycerate-3-phosp [file 40168_2022_1343_MOESM1_ESM.docx]

**Additional File 1**

**Title**

Metagenomics-resolved genomics provides novel insights into chitin turnover, metabolic specialization, and niche partitioning in the octocoral microbiome

**Authors**

Tina Keller-Costa^1,2*^, Lydia Kozma^1,3^, Sandra G. Silva^1,2^, Rodolfo Toscan^4^, Jorge Gonçalves^5^, Asunción Lago-Lestón^6^, Nikos C. Kyrpides^7^, Ulisses Nunes da Rocha^4^ & Rodrigo Costa^1,2,5*^

**Affiliations**

^1^Institute for Bioengineering and Biosciences, Department of Bioengineering, Instituto Superior Técnico, University of Lisbon, Lisbon, Portugal

^2^Associate Laboratory i4HB, Institute for Health and Bioeconomy, Instituto Superior Técnico, University of Lisbon, Lisbon, Portugal

^3^École Polytechnique Fédérale de Lausanne, Switzerland

^4^Helmholtz Centre for Environmental Research - UFZ, Leipzig, Germany

^5^Centro de Ciências do Mar (CCMAR), Universidade do Algarve, Faro, Portugal

^6^Centro de Investigación Científica y de Educación Superior de Ensenada, Ensenada, Mexico

^7^Department of Energy, Joint Genome Institute, Lawrence Berkeley National Laboratory, Berkeley, California, USA.

***Corresponding authors**

Institute for Bioengineering and Biosciences, Instituto Superior Técnico, University of Lisbon, Av. Rovisco Pais 1, Torre Sul, Office 11.6.11b, 1049-001 Lisbon, Portugal, Tel: (+351) 21 841 3167,

E-mail: [tinakellercosta@tecnico.ulisboa.pt](mailto:tinakellercosta@tecnico.ulisboa.pt); rodrigoscosta@tecnico.ulisboa.pt

**Detailed Methodology**

**Phylogenomics of the *Endozoicomonadaceae* and Ca. *Thioglobaceae* families**

The *Endozoicomonadaceae* phylogenomics assessment (Figure 2) included all (*N* = 29) genome assemblies publicly available on NCBI (National Center for Biotechnology Information, https: //www.ncbi.nlm.nih.gov), IMG/M (Integrated Microbial Genomes, [1]) and RAST (Rapid Annotation using Subsystem Technology, [2, 3]) by March 2021. The 29 genomes derived from cultured (i.e., isolates) and uncultured (MAGs and those obtained by single cell genomics - SAGs) bacteria. To root the tree, the genomes of five species of the closely related family *Hahellaceae* were included as an outgroup. The *Thioglobaceae* phylogenomics inference (Figure S1) included, next to the six octocoral-derived MAGs from this study, 33 publicly available genomes, MAGs and SAGs of the sulfur-oxidizing *Thioglobaceae* Arctic96 and SUP5 clades (three *Thioglobus* species, 16 *Thiomultimodus* species and divSUP05-5, as described in [4]). As an outgroup, 13 genomes of the ***Piscirickettsiaceae* family (order *Thiotrichales*) with representative type genomes of the genera *Thiomicrospira* (N=2),** *Thiomicrorhabdus* **(N=2)**, *Sulfurivirga* (N=1)*,* ***Methylophaga* (N=5)*,*** *Hydrogenovibrio* (N=2) and *Cycloclasticus* (N=1) were used. The ***Piscirickettsiaceae* family was** chosen as the outgroup since several ***Piscirickettsiaceae*** genomes were indeed identified by MiGA as the closest type species to our *Thioglobaceae* MAGs, albeit with low AAI values (see Table S1, Additional File 2 for details).

**Analysis of the chitinase gene sequence present on the *Endozociomonadaceae* MAGs**

Complete chitinase coding sequences (CDS) present on our MAGs were retrieved from RAST and translated into amino acid sequences, followed by Pfam annotations using the EMBOSS Transeq (<https://www.ebi.ac.uk/Tools/st/emboss_transeq>) and hmmscan (https://www.ebi.ac.uk/Tools/hmmer/search/hmmscan) algorithms of EMBL-EB. Phylogenetic inference of complete chitinase gene sequences was then performed following the methodology detailed in Raimundo et al., 2021 [5].

**Supplementary Figures**


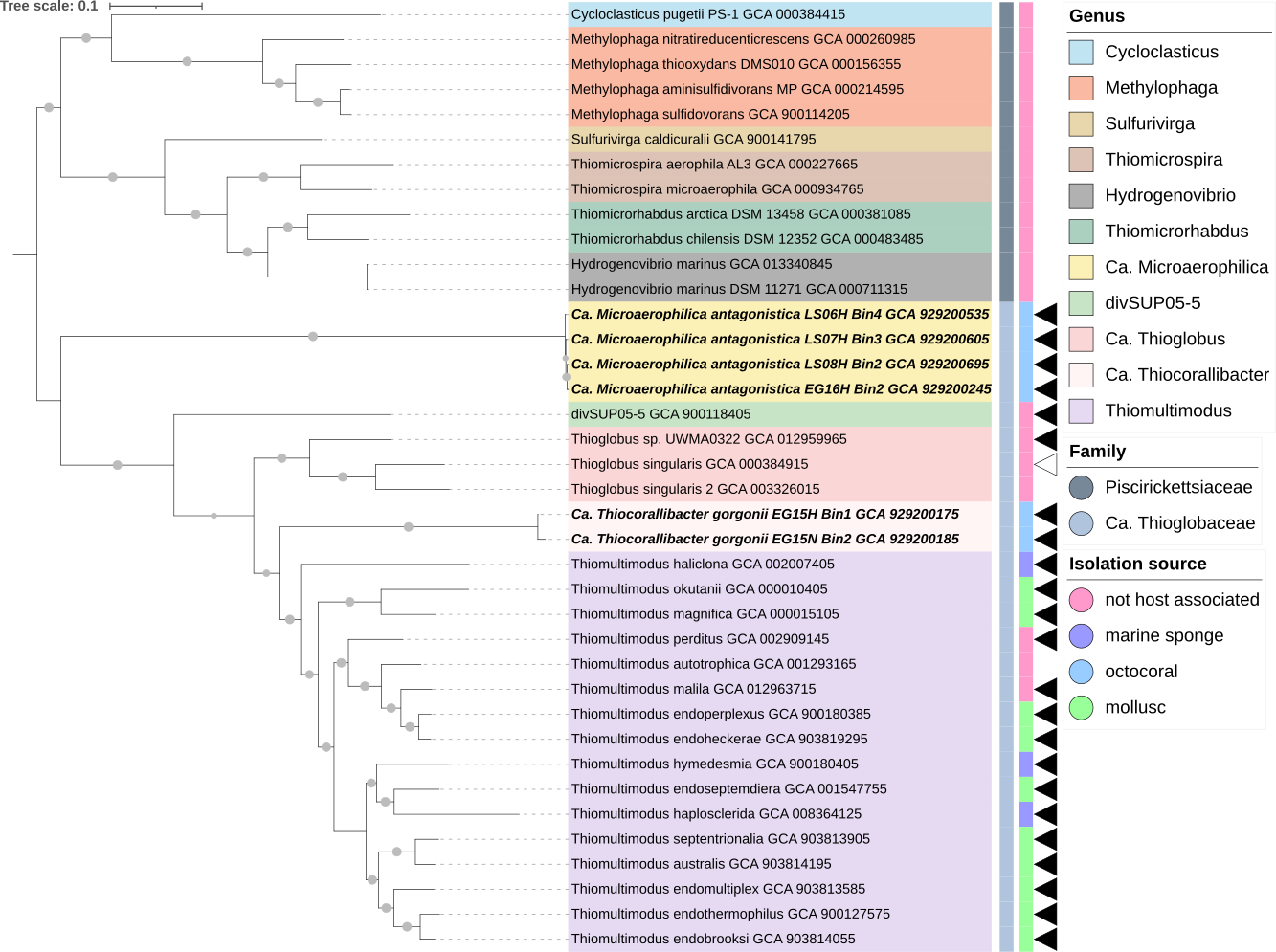


# **Figure S1.** Phylogenomic analysis of the Ca. *Thioglobaceae* family using SpeciesTreeBuilder v.01.0. Evolutionary history was inferred by using a Maximum likelihood method (FastTree2) based on alignment similarity of a set of 49 core, universal genes defined by COG (Clusters of Orthologous Groups of proteins) gene families. Grey dots on the branches indicate bootstrap support of >70%. Black triangles indicate a MAG, white triangles a SAG, the remaining genomes derived from isolates. The six Ca. *Thioglobaceae* MAGs (Ca. *Thiocorallibacter gorgonii* and Ca. *Microaerophilica antagonistica*) of this study are highlighted in bold-italics. All other Ca. *Thioglobaceae* genomes (*N*=20) were publicly available on NCBI. Assembly accession numbers are given next to the strain names. Twelve genomes of the related ***Piscirickettsiaceae*** family (*Thiotrichales, Gammaproteobacteria)* were used as an outgroup to root the tree. The inner coloured bar next to the tree shows family-level affiliation and the outer coloured bar the isolation source of the genomes. The tree is drawn to scale and was style-edited in iTOL.

###
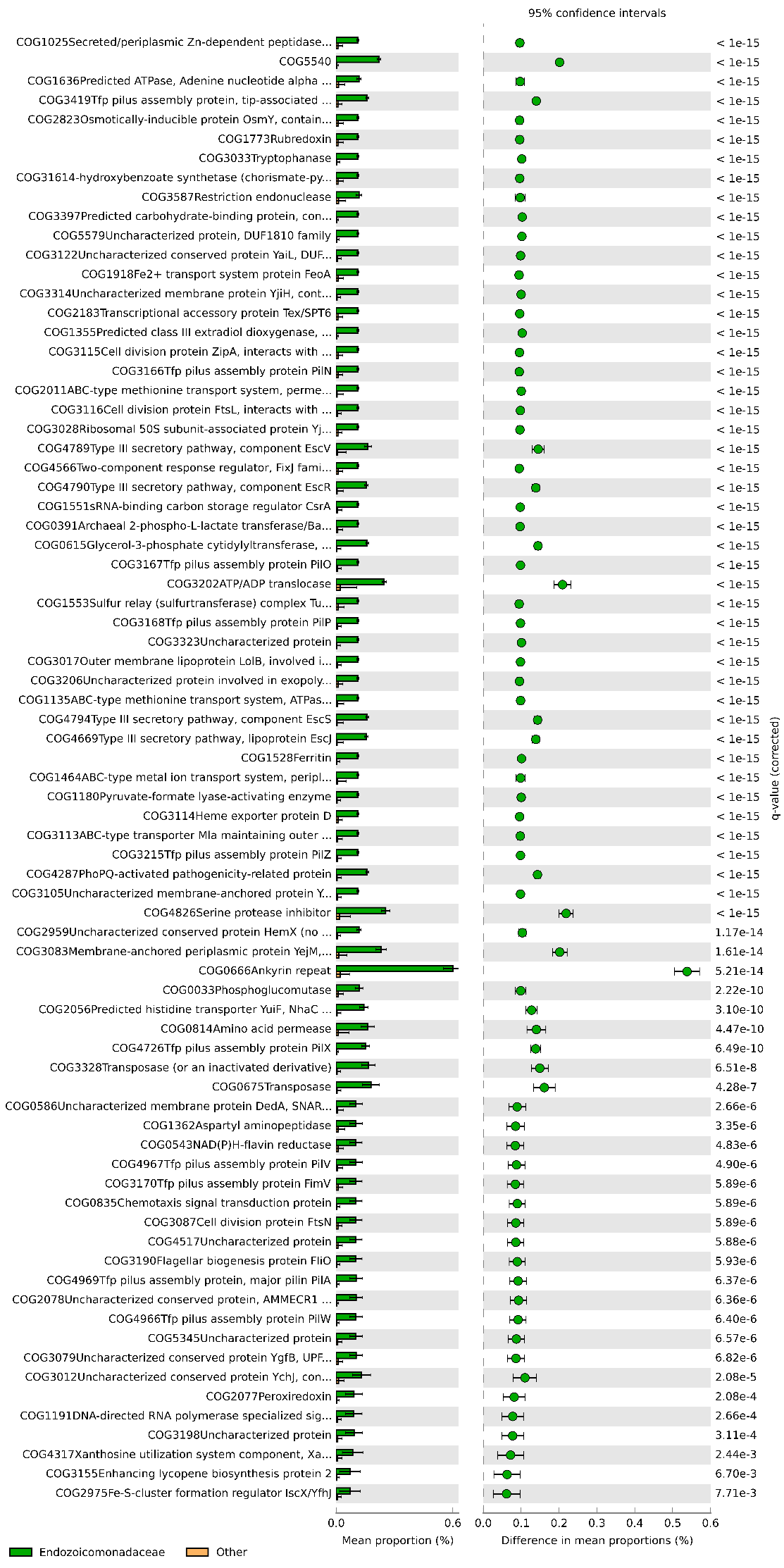


**Figure S2.**

COG functions (*N*=76) significantly enriched (*q*-value < 0.05) in the octocoral-derived Ca. *Gorgonimonas* (*Endozoico-monadaceae*) MAGs (*N*= 11, green), compared with all other MAGs (*N*=55) of this dataset. An one-sided, Welch’s t-test for unequal variances was performed with the STAMP (Statistical Analysis of Metagenomic Profiles) v.2.1.3 software. Multiple test correction was performed using the Benjamini-Hochberg correction (FDR). To further limit the displayed number of significant entries, an effect size filter was also applied, setting the “ratio of proportions” to 10.00.


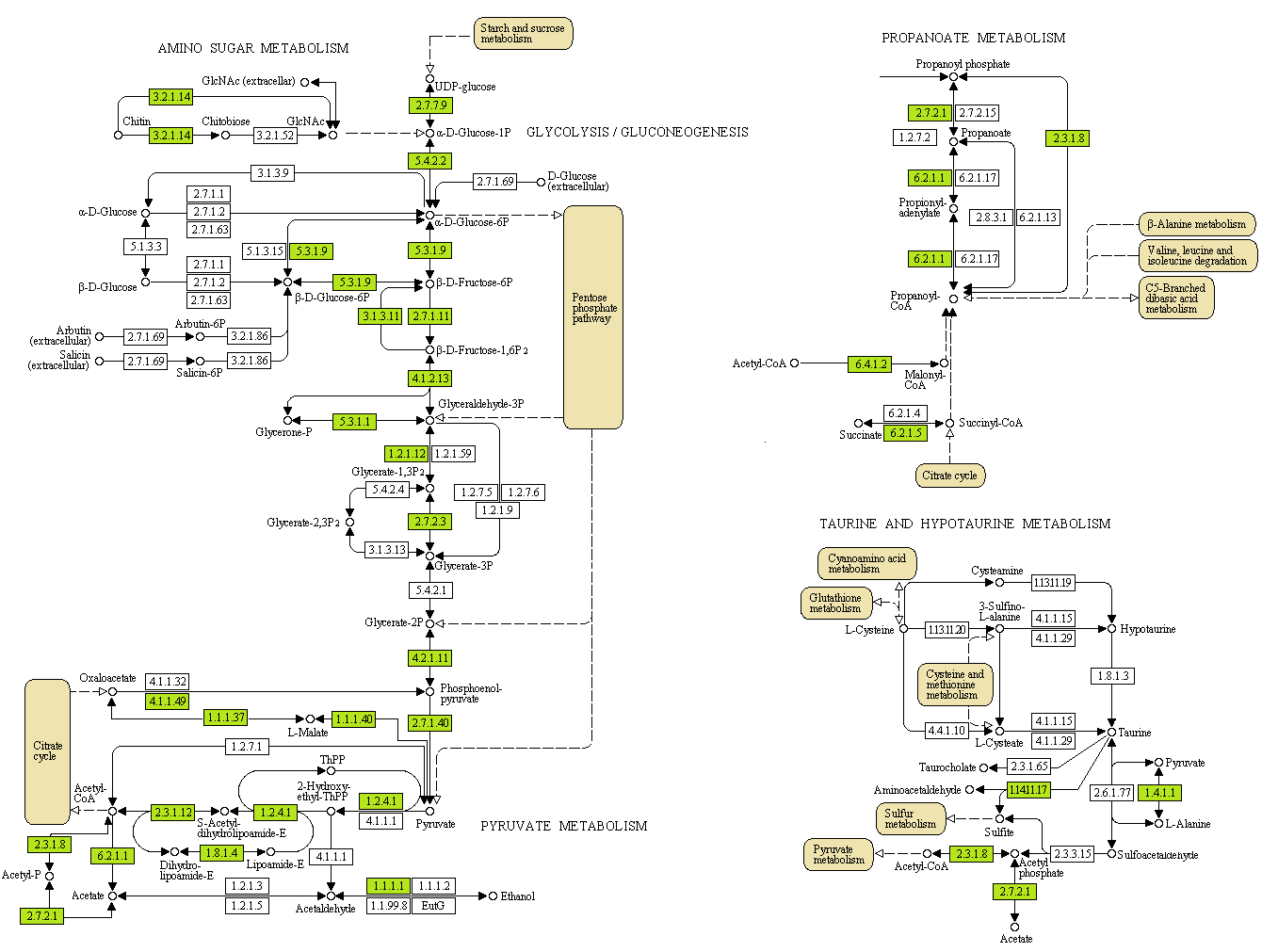


### Figure S3. KEGG metabolic pathway map of the Ca. *Gorgonimonas* (*Endozoicomonadaceae*) MAGs of this study. The metabolic map features glycolysis, amino-sugar, pyruvate, propanoate, and taurine metabolism in these symbionts. EC numbers of enzymes catalysing the reactions are given in rectangular boxes. EC numbers highlighted in green represent enzymes encoded on the Ca. *Gorgonimonas* MAGs. Beige boxes indicate connections to other metabolic pathways active in these MAGs.

###
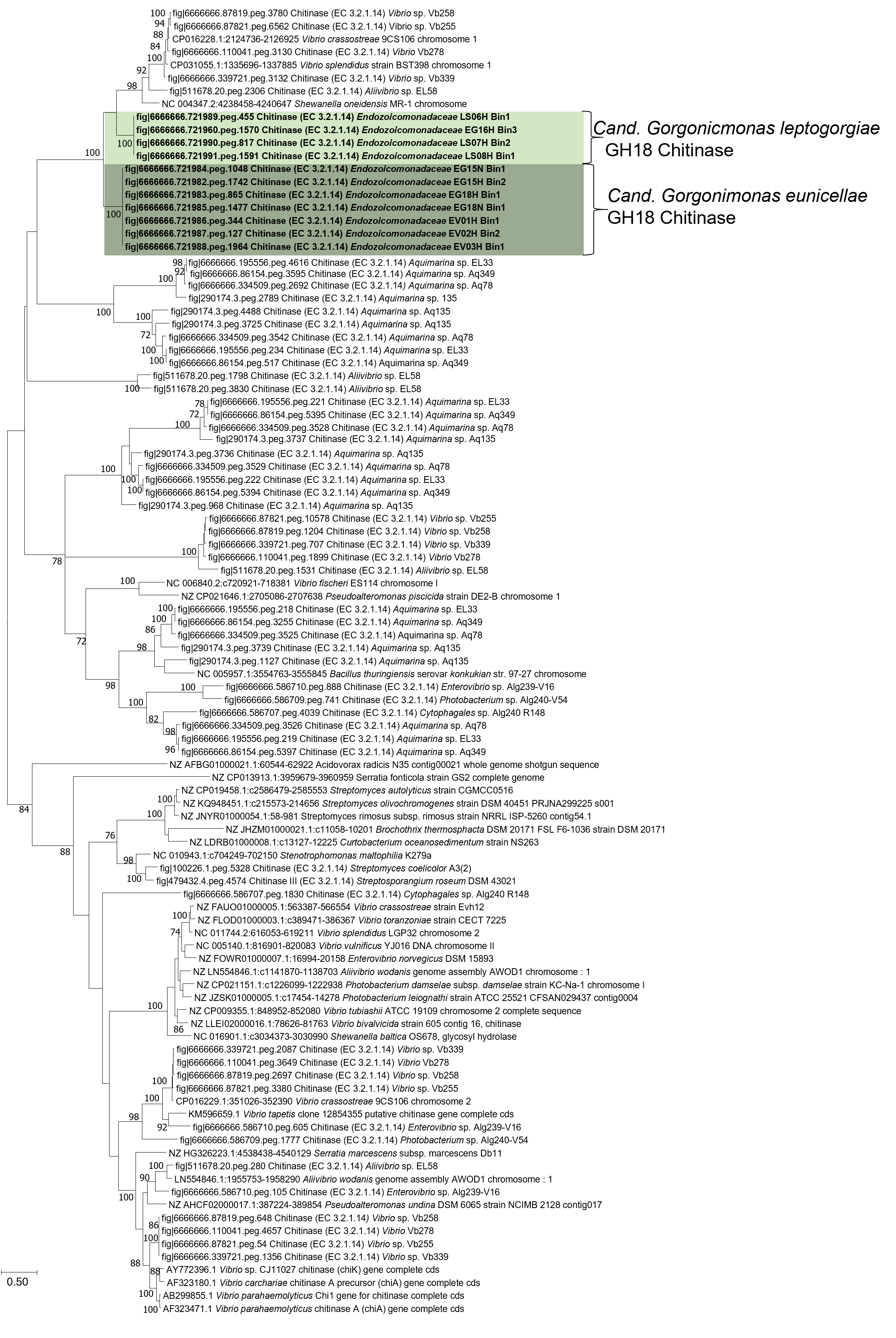


### Figure S4. Phylogeny of 101 full-length, bacterial endo-chitinase (EC 3.2.1.14) encoding genes, including the 11 endo-chitinase (GH18-family) genes from *Endozoicomonadaceae* MAGs (grey shadings) of this study. These 11 *Endozoicomonadaceae* chitinases form two distinct, species-specific clusters, whereby four genes belonged to the four MAGs of Ca. species *Gorgonimonas leptogorgiae* and were each 1731 bp (577 AA) long, carrying, besides a GH18 domain, two other domains of unknown function (DUF5011). The other seven genes belonged to the seven MAGs of Ca. species *G. eunicellae* and were each 1572 bp (524 AA) long, carrying the GH18 domain only. Closest neighbours are GH18-type chitinases of *Vibrionaceae* and *Shewanellaceae* (*Gammaproteobacteria*) isolates. The phylogeny was based on the Maximum Likelihood method and the General Time Reversible model (GTR+G+I). The tree with the highest log likelihood (-72263.15) is shown. The percentage of trees in which the associated taxa clustered together is shown next to the branches (50 repetitions). Codon positions included were 1st+2nd+3rd+Noncoding. All positions with less than 85% site coverage were eliminated. There were a total of 1206 nucleotide positions in the final dataset. More information about the other 90 bacterial chitinase sequences shown in this tree is available in the article of Raimundo et al. [5].

###
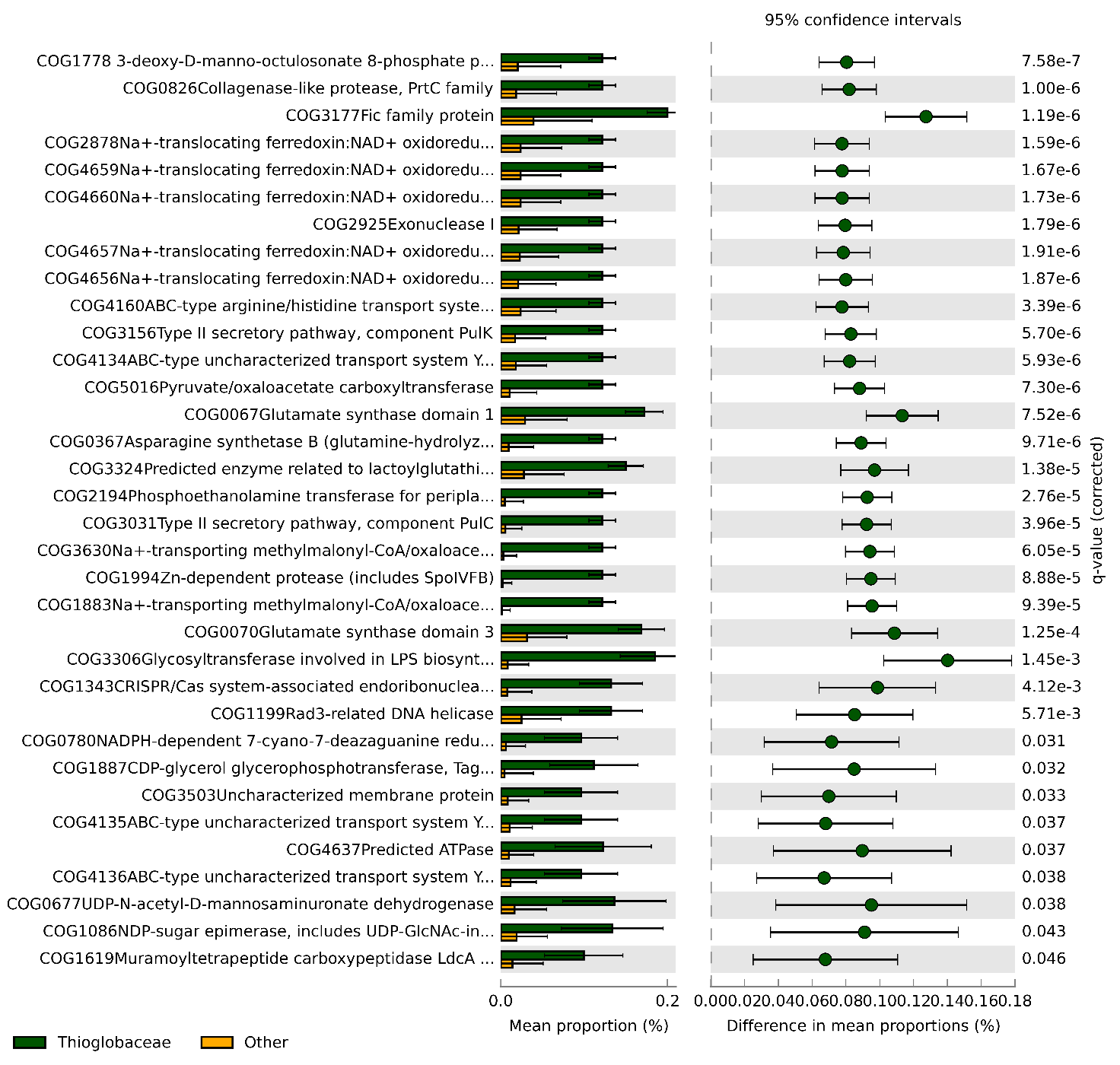


### Figure S5. COG functions (*N*=34) significantly enriched (*p*-value < 0.05) in the Ca. *Thioglobaceae* MAGs (*N*= 6, dark green), compared with all other MAGs (*N*=60) of this dataset. An one-sided Welch’s t-test for unequal variances was performed in STAMP v.2.1.3. Multiple test correction was performed using the Benjamini-Hochberg correction (FDR). To further limit the displayed number of significant entries, an effect size filter was also applied, setting the “ratio of proportions” to 5.00.


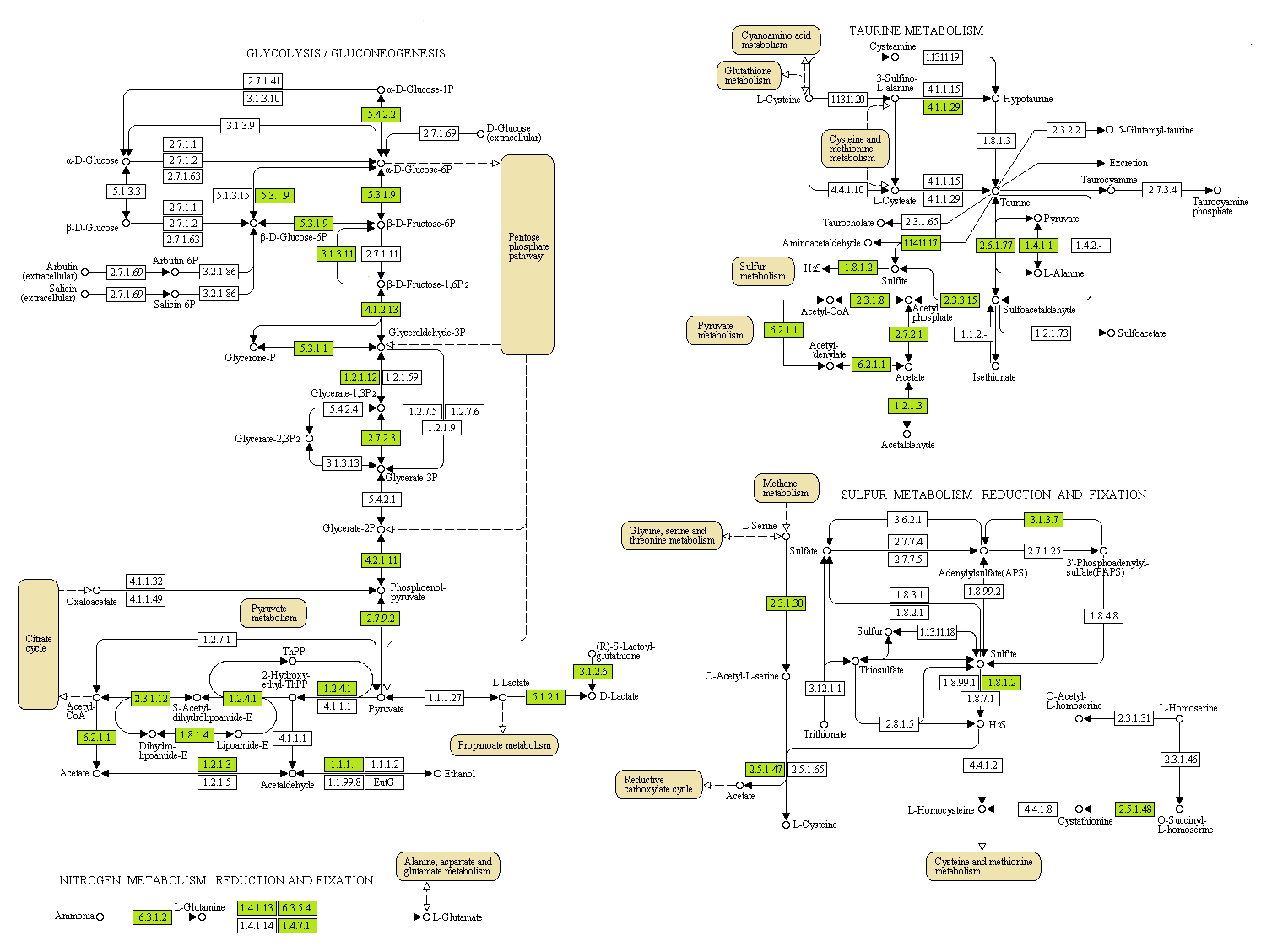


### Figure S6. KEGG metabolic pathway map of the Ca. *Thioglobaceae* MAGs of this study. The metabolic map features glycolysis, pyruvate, nitrogen, sulfur, and taurine metabolism. EC numbers of enzymes catalysing the reactions are given in rectangular boxes. EC numbers highlighted in green represent enzymes encoded on Ca. *Thioglobaceae* MAGs. Beige boxes indicate connections to other metabolic pathways active in these MAGs.


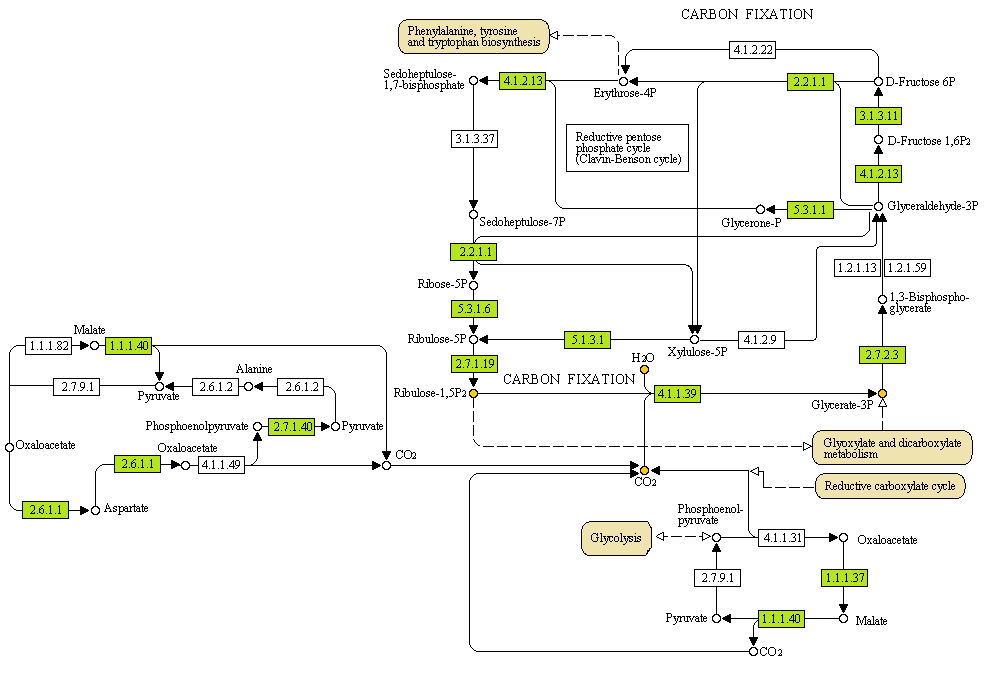


### Figure S7. KEGG carbon fixation map of Ca. *Thiocorallibacter gorgonii* MAG EG15H_Bin1 (Ca. *Thioglobaceae*). EC numbers of enzymes catalysing the reactions are given in rectangular boxes. EC numbers highlighted in green represent enzymes encoded on this MAG. Beige boxes indicate connections to other metabolic pathways active in these MAGs. Orange dots highlight the substrates (Ribulose-1,5-bisphosphate, CO_2_, and H_2_O) and product (2 Glycerate-3-phosphate, 2 H^+^) of Rubisco (EC 4.1.1.39).

### References

1. Chen IA, Chu K, Palaniappan K, Pillay M, Ratner A, Huang JH, Huntemann M, Varghese N, White JR, Seshadri R *et al*: IMG/M v.5.0: an integrated data management and comparative analysis system for microbial genomes and microbiomes. *Nucleic Acids Research* 2019, 8(47(D1)):D666-D677.

2. Overbeek R, Olson R, Pusch GD, Olsen GJ, Davis JJ, Disz T, Edwards RA, Gerdes S, Parrello B, Shukla M *et al*: The SEED and the rapid annotation of microbial genomes using subsystems technology (RAST). *Nucleic Acids Research* 2014, 42:D206–D214.

3. Aziz RK, Bartels D, Best AA, DeJongh M, Disz T, Edwards RA, Formsma K, Gerdes S, Glass EM, Kubal M *et al*: The RAST Server: rapid annotations using subsystems technology. *BMC Genomics* 2008, 9(75).

4. Ansorge R, Romano S, Sayavedra L, Rubin-Blum M, Gruber-Vodicka H, Scilipoti S, Molari M, Dubilier N, Petersen J: The hidden pangenome: comparative genomics reveals pervasive diversity in symbiotic and free-living sulfur-oxidizing bacteria. *bioRxiv* 2020, 12.11.421487.

5. Raimundo I, Silva R, Meunier L, Valente S, Keller-Costa T, Costa R: Functional metagenomics reveals differential chitin degradation and utilization features across free-living and host-associated marine microbiomes. *Microbiome* 2021, 9:43.
